# Synthetic Vesicles for Sustainable Energy Recycling and Delivery of Building Blocks for Lipid Biosynthesis†

**DOI:** 10.1021/acssynbio.4c00073

**Published:** 2024-04-18

**Authors:** Eleonora Bailoni, Miyer F. Patiño-Ruiz, Andreea R. Stan, Gea K. Schuurman-Wolters, Marten Exterkate, Arnold J. M. Driessen, Bert Poolman

**Affiliations:** ^‡^Department of Biochemistry, and ^§^Department of Molecular Microbiology, Groningen Biomolecular Sciences and Biotechnology Institute, Nijenborgh 4, 9747 AG Groningen, The Netherlands; ∥ Department of Membrane Biogenesis and Lipidomics, Institute of Biochemistry, Heinrich-Heine-Universität Düsseldorf, Universitätsstraβe 1, 40225 Düsseldorf, Germany

**Keywords:** ATP recycling, glycerol 3-P/Pi antiporter, out-of-equilibrium metabolic network, phospholipid biosynthesis, building block delivery, synthetic cells

## Abstract

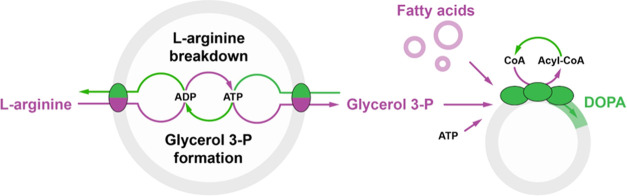

ATP is a universal energy currency that is essential for life. l-Arginine degradation via deamination is an elegant way to generate ATP in synthetic cells, which is currently limited by a slow l-arginine/l-ornithine exchange. We are now implementing a new antiporter with better kinetics to obtain faster ATP recycling. We use l-arginine-dependent ATP formation for the continuous synthesis and export of glycerol 3-phosphate by including glycerol kinase and the glycerol 3-phosphate/Pi antiporter. Exported glycerol 3-phosphate serves as a precursor for the biosynthesis of phospholipids in a second set of vesicles, which forms the basis for the expansion of the cell membrane. We have therefore developed an out-of-equilibrium metabolic network for ATP recycling, which has been coupled to lipid synthesis. This feeder–utilizer system serves as a proof-of-principle for the systematic buildup of synthetic cells, but the vesicles can also be used to study the individual reaction networks in confinement.

## Introduction

The bottom-up construction of living cells is one of the major challenges in synthetic biology. While an all-encompassing definition of life has not yet been formulated, it is universally agreed that maintaining an out-of-equilibrium state is a necessity for autonomous growth. Living cells fulfill this requirement by taking up nutrients and excreting waste products and by coupling metabolic energy to otherwise unfavorable reactions. Many of these essential processes (e.g., DNA replication, transcription/translation, lipid biosynthesis, uptake of nutrients, etc.) are fueled by the hub metabolite^1^ adenosine triphosphate (ATP), which is typically present in cells in millimolar concentration. The *de novo* synthesis of ATP involves the combination of ribose with adenine to form adenosine, and the stepwise phosphorylation of adenosine to form ATP. Bacteria that lack the ability to synthesize adenine nucleotides *de novo* must acquire ATP or ADP from the environment, e.g., the cytosol of the host cell. Nucleotide transporters with specificities for different nucleotide substrates are present in intracellular bacteria and organelles like mitochondria.^2,3^ In aerobically growing *Escherichia coli*, the ATP pool is turned over about 5 times per second.^3^ However, the vast majority of this ATP is not synthesized *de novo* or taken up by the cell but regenerated from its breakdown products, primarily ADP and inorganic phosphate (Pi). Therefore, any synthetic cell will require the design of a metabolic network for the regeneration or recycling of ATP.

**Figure 1:**
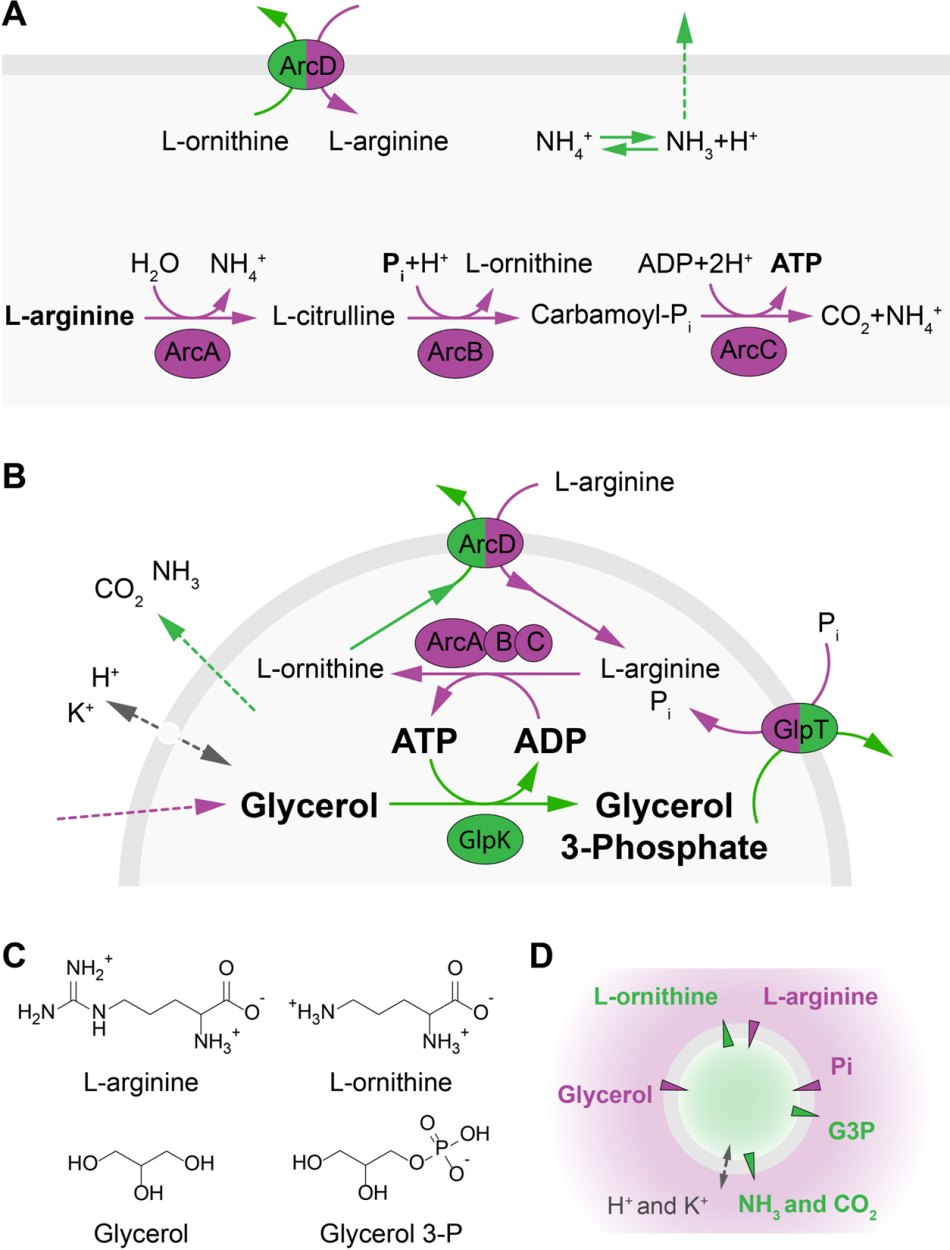
Synthetic pathway for ATP recycling and glycerol 3-phosphate formation. (A) Schematic of the l-arginine breakdown pathway (ArcABCD) that provides ATP; the side products (l-ornithine, CO_2_, and NH_4_^+^ in rapid equilibrium with NH_3_) are released into the external medium either by transport or passive diffusion through the phospholipid bilayer. (B) Schematic overview of out-of-equilibrium glycerol 3-phosphate synthesis and export fueled by l-arginine breakdown. A glycerol 3-phosphate/Pi antiporter (GlpT) allows for the exchange of internal glycerol 3-phosphate for the external Pi. (C) Chemical structures of l-arginine, l-ornithine, glycerol, and glycerol 3-phosphate. (D) Electrochemical gradients of solutes involved in l-arginine breakdown and glycerol 3-phosphate synthesis across the vesicle membrane. Proton, potassium, and ammonium gradients are dissipated by nigericin plus valinomycin (double-sided dashed arrow);^18^ ammonium is also in rapid equilibrium with ammonia, a small neutral molecule that passively diffuses through the phospholipid bilayer.^17^

ATP recycling has been successfully achieved in complex photosynthetic systems driven by a proton motive force (PMF) in a light-dependent manner.^4^ These artificial chloroplasts have been reconstituted into giant vesicles, mimicking organelles of eukaryotic cells, and used to power actin polymerization^5^ and protein synthesis.^6^ In another study, a minimal system for cellular respiration and energy regeneration was obtained by coreconstituting the mitochondrial complex I with an alternative oxidase and F_o_F_1_-ATPase to recycle ATP in a NADH-dependent fashion.^7^ An alternative to ATP synthesis by the complex processes of respiration and photophosphorylation is the exploitation of simple metabolic networks.^8−10^ The breakdown of l-arginine to ATP requires only four proteins: an l-arginine/l-ornithine antiporter and three metabolic enzymes (Figure 1a). The strict coupling of l-arginine uptake to the excretion of the end product l-ornithine forms the basis for maintaining out-of-equilibrium conditions. As the formed CO_2_ and NH_3_ leave the cell by passive diffusion across the membrane, no side products remain. ATP formation from l-arginine breakdown has been used to drive solute uptake and volume homeostasis in a reconstituted system^9^ and the synthesis of glycerol 3-phosphate, an essential precursor of phospholipids.^10^

The simplest phospholipid is phosphatidic acid (PA), a central precursor of, e.g., phosphatidylethanolamine (PE), phosphatidylglycerol (PG), phosphatidylserine (PS), phosphatidylcholine (PC), and cardiolipin (CL). PA formation requires the sequential transfer of two acyl chains to glycerol 3-phosphate by acyltransferases, which in eukaryotic cells occurs on the cytosolic leaflet of the endoplasmic reticulum.^11,12^ We previously reconstituted the *E. coli* acyltransferases PlsB and PlsC together with FadD, a soluble enzyme that catalyzes acyl-CoA formation in the presence of ATP plus coenzyme A,^13^ which led to significant membrane expansion starting from a free fatty acid feed. Additionally, PA formation has been demonstrated from genetically encoded *plsB* and *plsC*,^14^ a fatty acid formation module^15^ and from a minigenome encoding the components for headgroup functionalization.^16^

We now present a kinetically more favorable version of the l-arginine breakdown pathway for ATP recycling, which fuels the intravesicular production of the lipid precursor glycerol 3-phosphate. A glycerol 3-phosphate/Pi antiporter^10^ enables (i) export of the synthesized glycerol 3-phosphate; (ii) recycling of phosphate; and (iii) continued synthesis of ATP by the l-arginine breakdown pathway. Overall, nonequilibrium conditions are maintained by the coupling of substrate import and product export via l-arginine/l-ornithine and glycerol 3-phosphate antiport. The continuous production of glycerol 3-phosphate enables lipid formation in a second set of vesicles. We thus couple modules for ATP recycling and lipid synthesis present in distinct compartments, allowing for control of the individual pathways. This feeder–utilizer setup includes advanced metabolic modules that can be expanded or used in combination with other reaction networks, either as independent vesicles or as synthetic organelles within larger vesicles.

## Results

### Glycerol 3-Phosphate/Pi Exchange to Overcome Phosphate Depletion

While gene-encoded components are ultimately needed for an autonomous synthetic cell, reconstitution in vesicles allows the combination of complex metabolic pathways, quantitatively characterizing them under controlled conditions and discovering emergent behavior of reaction networks.

We have previously combined ATP recycling by l-arginine breakdown with the synthesis of glycerol 3-phosphate, an essential phospholipid precursor. Glycerol can freely diffuse across the membrane and does not require a membrane transporter.^17^ However, when glycerol 3-phosphate is synthesized inside the vesicles by the glycerol kinase GlpK, the l-arginine breakdown pathway gets depleted of inorganic phosphate, ultimately limiting ATP recycling.^10^ To overcome this limitation, we expanded the glycerol 3-phosphate synthesis module with a component for glycerol 3-phosphate export and inorganic phosphate import. Hence, we overexpressed, purified, and coreconstituted GlpT from *E. coli* (Figure 1b,c).

GlpT is a secondary transporter of the major facilitator superfamily (MFS)^19^ that mediates glycerol 3-phosphate/Pi exchange.^20,21^ GlpT operates bidirectionally, and the direction of antiport depends on the sign of the total electrochemical potential. Thus, transport is independent of the orientation of the protein in the membrane. In our setup, GlpT facilitates the export of the internally formed glycerol 3-phosphate and the import of Pi from the external solution (Figure 1d). Noteworthily, as the external volume is more than two orders of magnitude larger than the cumulative vesicle lumen, we assume a constant external Pi concentration throughout the duration of the experiment. Hence, this expanded metabolic network represents a genuine out-of-equilibrium system where the phospholipid bilayer confines the recycling of ATP and glycerol 3-phosphate formation while allowing for all substrates to be externally sourced and all products to either be internally recycled or leave the system.

### Selection and Activity Screening of Homologues for Faster l-Arginine/l-Ornithine Exchange

The l-arginine/l-ornithine antiporter ArcD2 from *Lactococcus lactis* has significantly lower *k*_cat_ values (<1 s^–1^) than the water-soluble enzymes (ArcA = 3.3 s^–1^; ArcB, backward reaction = 410 s^–1^; ArcC1 = 200 s^–1^), suggesting that the transport step limits l-arginine breakdown in confinement.^8^ To overcome this kinetic bottleneck, we took a functional genomics approach to identify candidate homologues that can replace *L. lactis* ArcD2. We selected *arcD* genes with an amino acid identity of 30–60% with respect to the original antiporter (ArcD2 from *L. lactis*), which allows for significant variation, while the l-arginine/l-ornithine antiport function is likely retained (Figure S1). We also included *arcE* sequences, encoding putative l-arginine/l-ornithine antiporters that evolved independently from those of the ArcD family.^22,23^ We selected 10 putative ArcD- and ArcE-like proteins from the genomics approach, further guided by published biochemical data (Table S1, Figures S2, and S3).

The *arcD* and *arcE* genes were cloned into nisin A-based expression vectors and flanked by a 10× His-tag at either the N- or the C-terminus. The constructs were transformed into *L. lactis* JP9000 Δ*arcD1*Δ*arcD2*^24^ and the expression was optimized. Immunoblots showed the highest expression levels with ArcD from *Clostridium autoethanogenum*, *Lactobacillus sakei*, and *Pseudomonas aeruginosa* and ArcE from *Streptococcus pneumoniae*. All of these proteins were tested for l-arginine transport activity *in vivo* (Figure S4). We found that ArcD (with a N-terminal His-tag) from *L. sakei* had the highest rate of uptake. By assuming a similar level of expression as the original ArcD2, ArcD from *L. sakei* would have a 10-fold higher turnover number and was therefore chosen for further *in vitro* characterization (Table S4).

### Purification and Characterization of *L. sakei* ArcD

**Figure 2:**
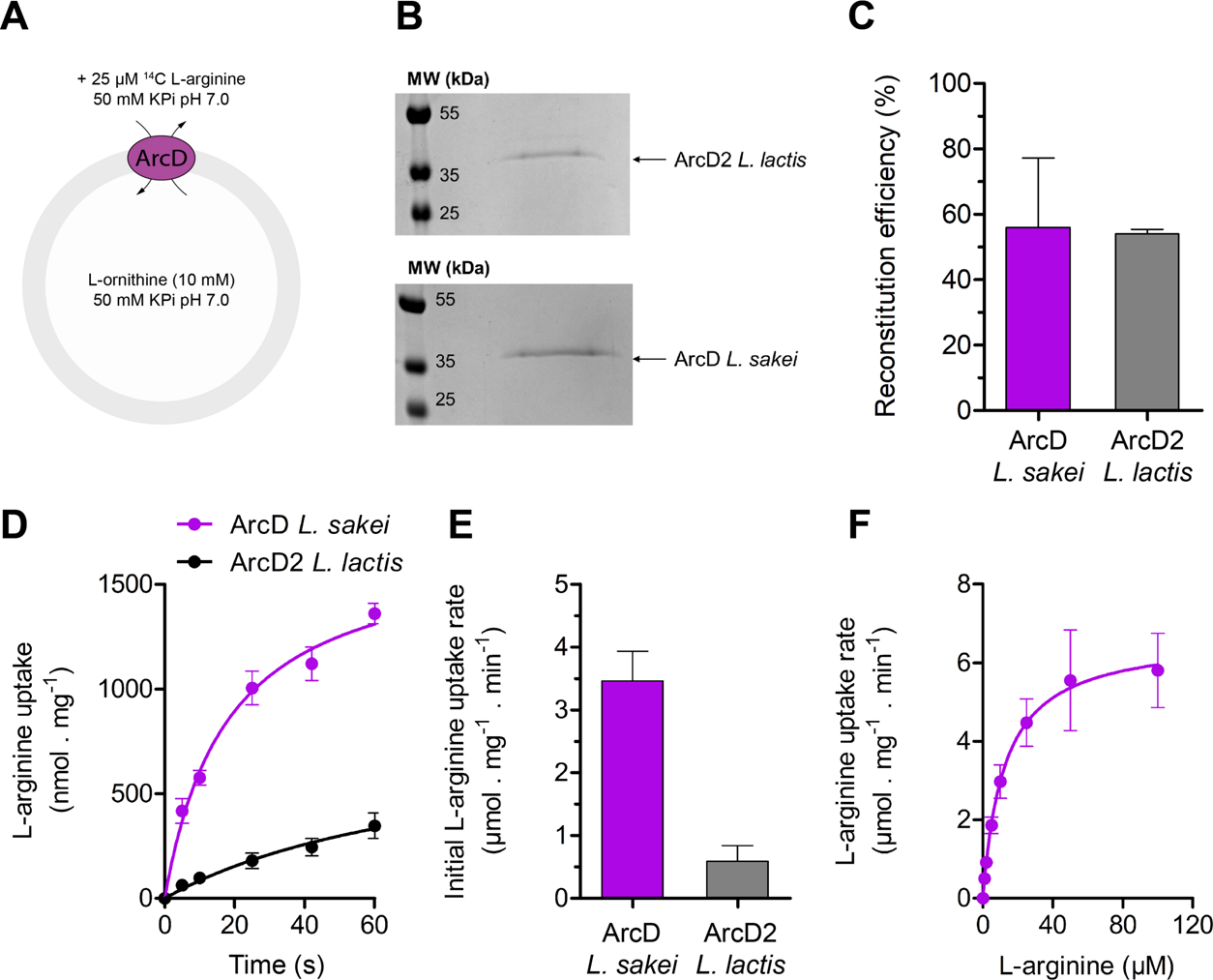
Characterization of *L. sakei* ArcD. (A) General scheme and conditions to determine l-arginine/l-ornithine transport activity in proteoliposomes composed of DOPE/DOPG/DOPC at 25:25:50 mol %. (B) SDS-PAA gel image of proteoliposomes with reconstituted ArcD from *L. sakei* (54 kDa) or ArcD2 from *L. lactis* (57 kDa).^8^ (C) Efficiency of the incorporation of *L. sakei* ArcD and *L. lactis* ArcD2 into liposomes, estimated by comparison of the band intensities with those of the purified proteins (*n* = 2 independent reconstitutions, error bars are s.e.m.). (D) Uptake of l-arginine in *L. lactis* ArcD2 and *L. sakei* ArcD (*n* = 5 independent reconstitutions, error bars are s.e.m.) reconstituted in liposomes with a lipid-to-protein ratio of 400:1 w/w and loaded with 10 mM l-ornithine. The external l-arginine concentration was 25 μM ^14^C-l-arginine. Accounting for the reconstitution efficiency, initial transport rates of 0.6 ± 0.1 μmol·mg protein^–1^ min^–1^ (0.5 ± 0.1 s^–1^) and 3.5 ± 0.2 μmol.mg protein^–1^ min^–1^ (3.0 ± 0.2 s^–1^) were obtained for *L. lactis* ArcD2 and *L. sakei* ArcD, respectively. (E) Comparison of initial rates of l-arginine uptake for *L. sakei* ArcD and *L. lactis* ArcD2 (*n* = 5 independent reconstitutions, error bars are s.e.m.), calculated from uptake curves as shown in panel (D). (F) External l-arginine concentration dependence of the initial rate of uptake for *L. sakei* ArcD reconstituted in vesicles loaded with 10 mM l-ornithine (*n* = 2 independent reconstitutions, error bars are s.e.m.). A continuous line represents the fitting of the Michaelis–Menten equation to the experimental data.

ArcD from *L. sakei* was purified and reconstituted in liposomes composed of DOPE/DOPG/DOPC at 25:25:50 mol %. ArcD2 and ArcD from *L. sakei* had similar reconstitution efficiencies (∼60%), as estimated by SDS-PAA gel electrophoresis (Figures 2a–c and S5). A random 50% orientation of the l-arginine/l-ornithine antiporter is assumed for the reconstitution. A scrambled membrane protein orientation does not compromise the pathway activity, as the antiport is governed by the metabolite gradients (Figure 1d), and directional l-arginine import and l-ornithine export are ensured under our experimental conditions.

The transport activity was measured via the uptake of radiolabeled l-arginine (25 μM) in exchange for internal l-ornithine. Accounting for the protein amount, an initial transport rate of 3.5 ± 0.2 μmol mg protein^–1^ min^–1^ (3.0 ± 0.2 s^–1^) was determined for *L. sakei* ArcD, a value ∼5-fold higher than what was found for *L. lactis* ArcD2 (0.6 ± 0.1 μmol mg protein^–1^ min^–1^, 0.5 ± 0.1 s^–1^; Figure 2d,e). By variation of the external concentration of l-arginine, the affinity constant (*K*_M_) and turnover number (*k*_cat_) were determined for the *L. sakei* antiporter (Figure 2f and Table S5). In agreement with the initial transport rates, the turnover number of *L. sakei* ArcD is much higher than that of *L. lactis* ArcD2, while the *K*_M_ values are similar.^8^

### ATP Formation

**Figure 3:**
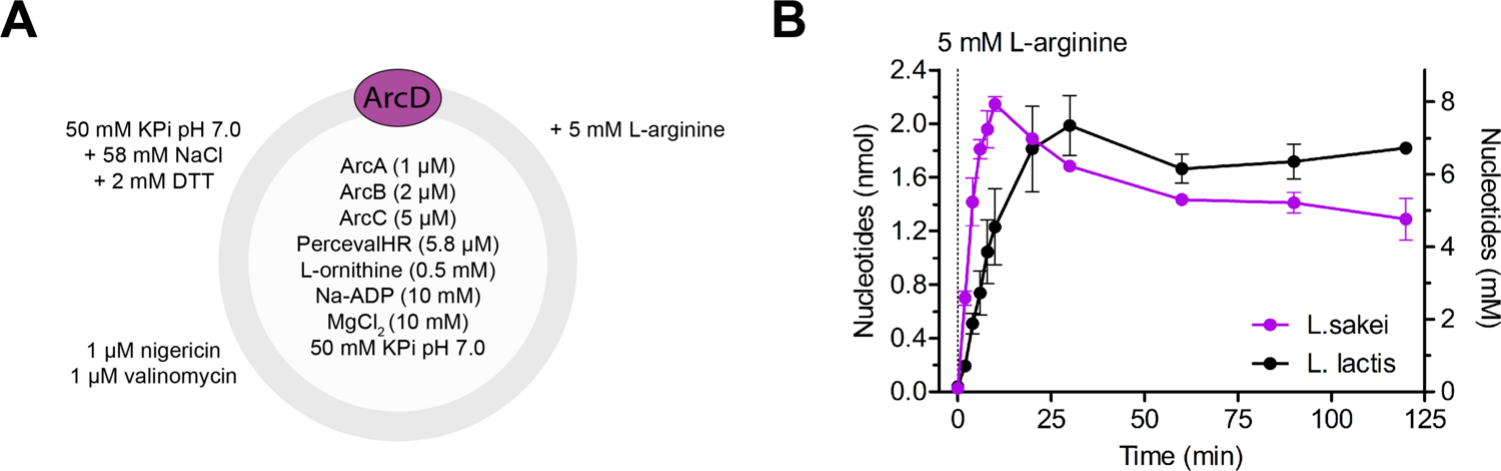
ATP formed by the reconstituted l-arginine breakdown pathway with *L. sakei* ArcD or *L. lactis* ArcD2. (A) Schematic of the vesicle system. ArcD from *L. sakei* or ArcD2 from *L. lactis* was reconstituted in liposomes composed of DOPE, DOPG, and DOPC at 25:25:50 mol % with a 400:1 w/w lipid-to-protein ratio. (B) ATP levels were quantified by chemiluminescence (*n* = 3 independent reconstitutions; error bars are s.e.m.). The vesicles with *L. sakei* ArcD have a ∼2.5× higher initial rate of ATP formation than those with *L. lactis* ArcD2.

If l-arginine/l-ornithine exchange would be fully rate-limiting for ATP production, then an increase in the rate of transport should lead to a corresponding increase in the rate of ATP synthesis. To test this hypothesis, we equipped vesicles composed of DOPE/DOPG/DOPC (25:25:50 mol %), containing either *L. lactis* ArcD2 or *L. sakei* ArcD, with the components of the l-arginine breakdown pathway^10^ (Figure 3a) and we quantified the ATP formation rate by chemiluminescence. From the linear increment during the first 5 min of l-arginine metabolism, we find that the ATP synthesis is ∼2.5-fold faster with ArcD from *L. sakei* than with ArcD2 from *L. lactis* (Figure 3b), i.e., under conditions that the difference in the initial l-arginine uptake rate is ∼5-fold (Figure 2d). Similar results were obtained when ATP formation was followed with the fluorescent ATP/ADP sensor PercevalHR (Figure S6). We thus conclude that l-arginine/l-ornithine antiport is not the sole kinetically limiting factor, and additional bottlenecks must be present in the pathway. A likely candidate is the reaction catalyzed by the carbamoyl transferase ArcB, which at pH 7.0 has an equilibrium constant of 1.2 × 10^5^ in the direction of l-citrulline formation^8^ (Figure 1a). Hence, high concentrations of l-citrulline and inorganic phosphate and low levels of products are required for a net reaction in the desired direction, i.e., ornithine plus carbamoyl phosphate formation.

### Coreconstitution of ArcD and GlpT in LUVs

**Figure 4:**
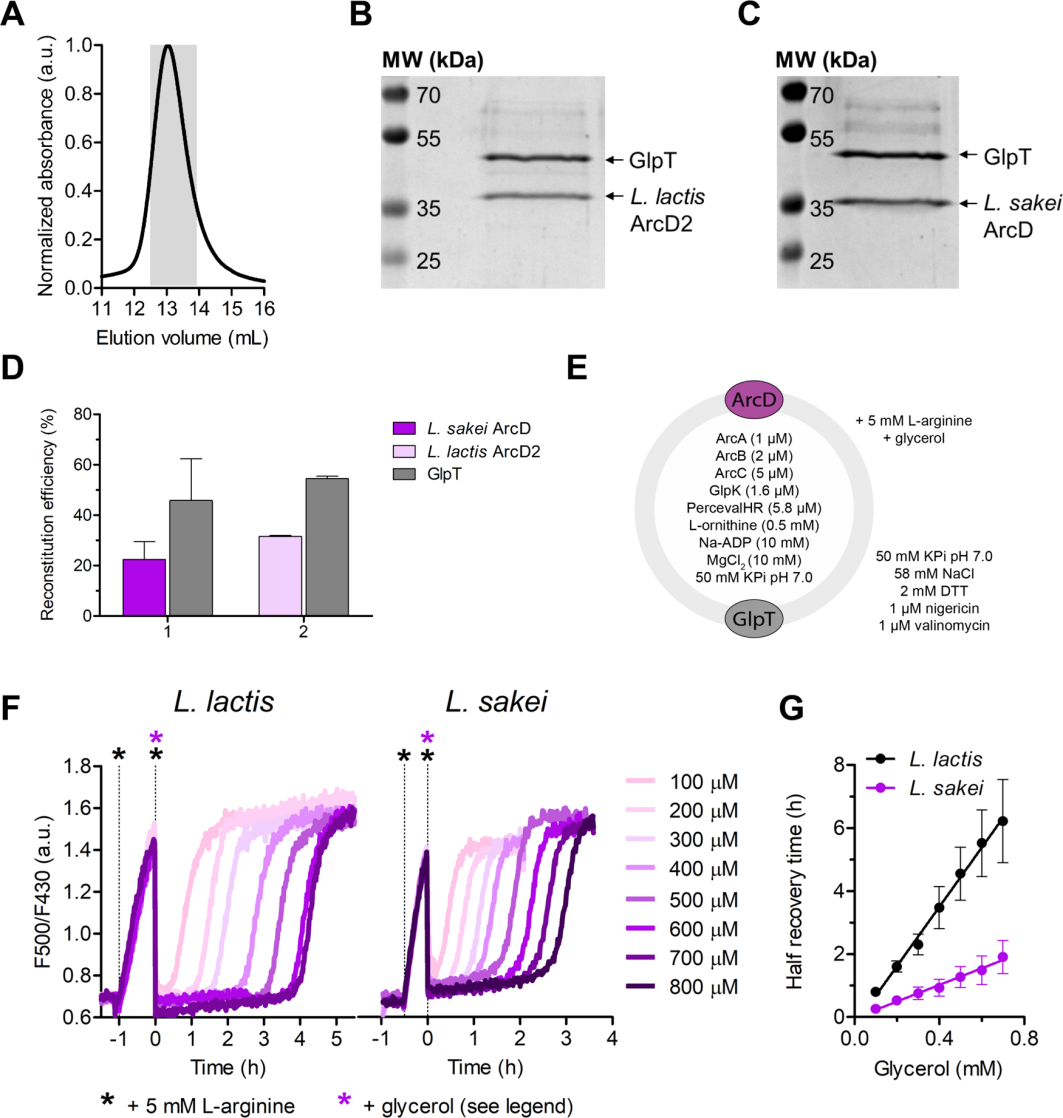
ATP/ADP ratio coupled to the synthesis and export of glycerol 3-phosphate. (A) Size-exclusion chromatogram of purified *E. coli* GlpT. Elution fractions 12.5–14.0 mL were collected and used for coreconstitution. (B, C) SDS-PAA gel images of proteoliposomes containing coreconstituted *E. coli* GlpT (54 kDa^25^) plus *L. lactis* ArcD2 (57 kDa,^8^ panel (B)) or *L. sakei* ArcD (54 kDa, panel (C)). (D) Efficiency of the coincorporation of *L. sakei* ArcD (1) or *L. lactis* ArcD2 (2) plus *E. coli* GlpT in vesicles, estimated from the intensities of protein bands on SDA-PAA gels (*n* = 2 independent reconstitutions, error bars are s.e.m.). (E) Schematic of the vesicle system. ArcD2 from *L. lactis* or ArcD from *L. sakei* was reconstituted together with GlpT from *E. coli*. (F) ATP/ADP ratio over time in the presence of ArcD2 from *L. lactis* (left panel) or ArcD from *L. sakei* (right panel); traces are representative of at least 3 independent coreconstitutions. ATP generation was initiated by supplying 5 mM l-arginine at *t* = −1 for *L. lactis* and *t* = −0.5 for *L. sakei*. Next, glycerol 3-phosphate synthesis was initiated at *t* = 0 by adding increasing concentrations of glycerol (0–0.7 mM). Additional 5 mM l-arginine was added at *t* = 0 and at 3–4 h intervals (not indicated). (G) Half-recovery time as a function of glycerol as an estimate of the glycerol 3-phosphate formation rate (*n* = 4 and *n* = 3 independent reconstitutions for *L. lactis* and *L. sakei*, respectively; error bars are s.e.m.). Ensemble half-recovery times (dt/d[glycerol]) were obtained from linear regression fits (*L. lactis*: 9.40 ± 1.32 h/mM glycerol; *L. sakei*: 2.64 ± 0.53 h/mM glycerol).

Next, we overexpressed and purified *E. coli* GlpT^25^ by affinity and size-exclusion chromatography (Figure 4a) and coreconstituted the protein with either *L. lactis* ArcD2 or *L. sakei* ArcD in vesicles composed of DOPE/DOPG/DOPC at 25:25:50 mol %. We expected that the rates of glycerol 3-phosphate formation and export would be faster than those of l-arginine breakdown, given the kinetic parameters of the enzymes and transporters. In fact, the synthesis of glycerol 3-phosphate by GlpK depletes the internal preformed ATP pool generated by l-arginine breakdown in less than 1 min.^10^ In addition, the *k*_cat_ of *E. coli* GlpT is higher than that of the l-arginine/l-ornithine antiporters (Table S5). Thus, we used a 1:1 mol ratio of GlpT/ArcD, at a lipid-to-protein ratio of 400:1 w/w for each protein (Figure 4b,c). Approximately 60% of GlpT was inserted into the vesicles. However, the reconstitution efficiency of both ArcD variants dropped to ∼30% in the presence of GlpT (Figure 4d). As for ArcD, the orientation of GlpT is not important because the antiporter is functional in both directions, and the direction of transport is determined by the glycerol 3-phosphate and inorganic phosphate gradients. In our setup, only net glycerol 3-phosphate export and phosphate import are possible (Figure 1d).

### Kinetics and Robustness of ATP-Driven Glycerol 3-Phosphate Synthesis and Export

Glycerol 3-phosphate formation coupled to glycerol 3-phosphate/Pi exchange represents an ideal load to study the performance of the l-arginine breakdown pathway in terms of kinetics (e.g., ATP recycling rates with different ArcD homologues, stability over time) and robustness (e.g., ATP equivalents formed). We encapsulated the l-arginine breakdown pathway in vesicles reconstituted with GlpT plus ArcD2 from *L. lactis* or ArcD from *L. sakei*, together with soluble GlpK and the ATP/ADP sensor PercevalHR (Figure 4e), and we determined the ATP/ADP ratio over time (Figures 4f and S7). We allowed an initial cycle of ATP formation by the addition of 5 mM l-arginine. When the ATP/ADP plateau was reached, we initiated glycerol 3-phosphate formation and export by adding glycerol in concentrations ranging from 0.1 to 0.7 mM. At this time, an additional 5 mM l-arginine was added (and hereafter at 3–4 h time intervals) to ensure that ATP recycling would not be limited by l-arginine depletion in the external medium.^9^ Glycerol rapidly enters the vesicles by passive diffusion,^17^ and glycerol 3-phosphate formation and export are faster than ATP recycling. In agreement, we observed an instantaneous drop in the ATP/ADP ratio upon glycerol addition. The ATP/ADP signal recovers when all of the glycerol has been converted into glycerol 3-phosphate. The time until recovery is a linear function of the glycerol concentration, while the rate of ATP/ADP recovery upon glycerol depletion is similar in all cases (Figure 4f).

Strikingly, the coreconstitution of the glycerol 3-phosphate/Pi antiporter allowed ATP/ADP recovery at glycerol concentrations well beyond what is possible on the basis of the levels of inorganic phosphate in the vesicle lumen, demonstrating that the internal Pi pool is replenished efficiently. We calculated the amounts of ATP produced from the amount of glycerol metabolized and the effective volumes of the vesicles and outside medium, which correspond to 14 rounds of ATP recycling inside the vesicles. These results showcase the robustness of l-arginine breakdown when coupled to a reaction load. The system performs multiple cycles of ATP regeneration that are only limited by the availability of external substrates (here, l-arginine, glycerol, and Pi) and the elimination of end products (l-ornithine, glycerol 3-phosphate, NH_3_, and CO_2_), illustrating the sustainable power of out-of-equilibrium reaction networks.

We then asked how many molecules of ATP are generated on average per vesicle in the time frame of the experiment. A glycerol concentration of 0.7 mM in a 120 μL reaction volume corresponds to 84 nmol or a total of 5.1 × 10^16^ glycerol equivalents. Hence, the same number of ATP molecules must have been generated by l-arginine breakdown. The total internal vesicle volume in the 120 μL reaction volume is 0.875 μL^10^ and corresponds to approximately 1.8 × 10^10^ vesicles with an average radius of 226 nm and an average volume of 0.05 fL.^9^ Thus, on average, 2.8 × 10^6^ ATP equivalents are converted per vesicle over a time period of 3 (*L. sakei* ArcD) to 5 (*L. lactis* ArcD2) hours. This ATP production rate allows DNA replication, DNA transcription, nutrient transport, cell division, and other processes to be executed in the vesicles, but it is insufficient for full growth and division of a synthetic cell with a doubling time of a few hours.^26^

To compare the l-arginine breakdown rate between the two different ArcD variants, we plotted the half-recovery time (i.e., the time when half of the ADP pool had been converted back into ATP) of each ATP/ADP trace as a function of the glycerol concentration (Figure 4g). We see that the ATP/ADP signal recovers 2- to 3-fold faster in vesicles with ArcD from *L. sakei* than ArcD2 from *L. lactis*, which is in line with the 2.5-fold increase in the ATP synthesis rate. From the estimated ATP requirement for a synthetic cell cycle (3.6 × 10^8^ equivalents)^26^ and the measured half-recovery rate (Figure 4g), we calculate that a synthetic cell of 400 nm containing the here presented, improved l-arginine breakdown pathway would support a cell cycle with a doubling time of ∼10 days.

### Design of a Feeder–Utilizer Vesicle System

**Figure 5:**
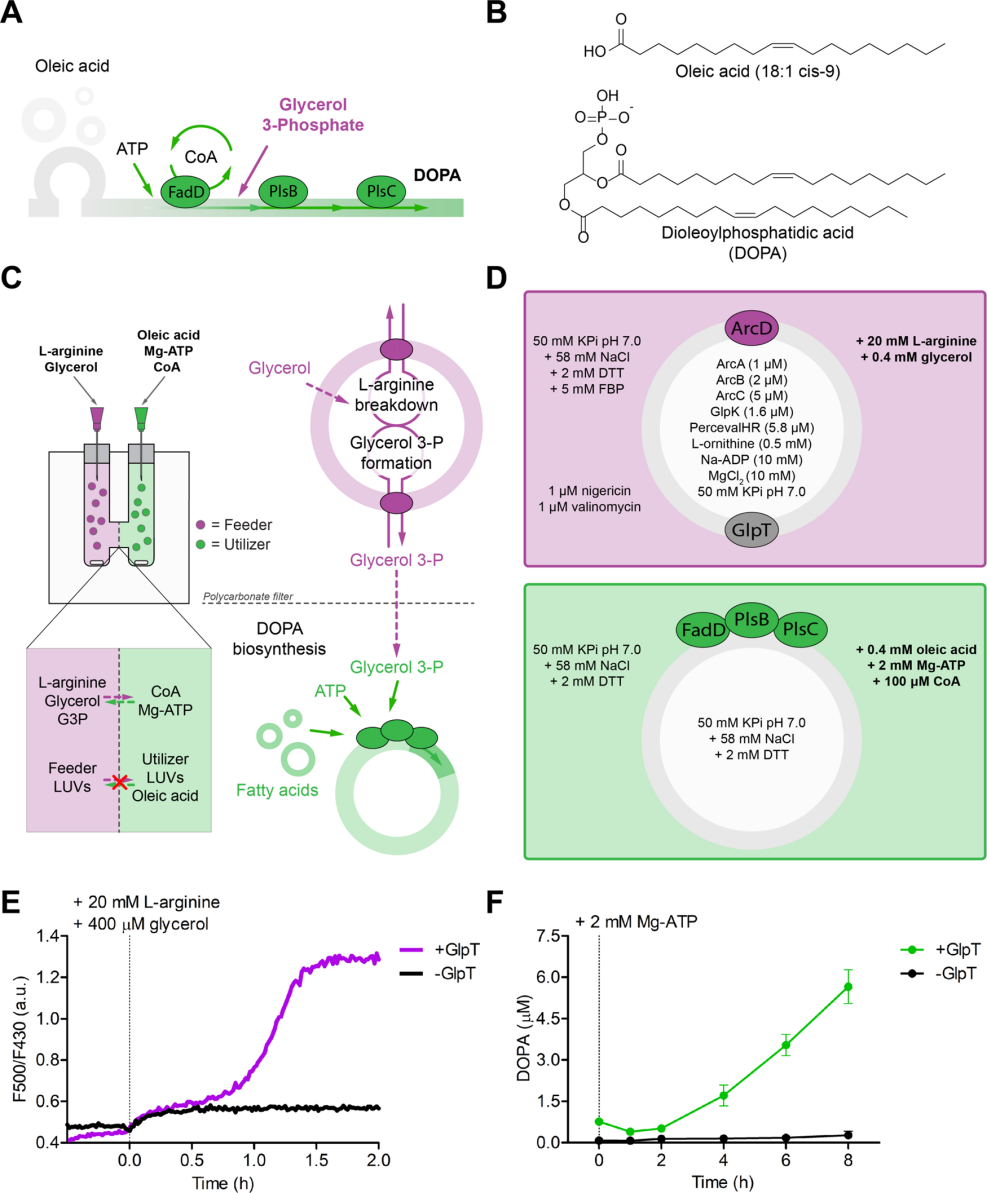
Out-of-equilibrium synthesis of phospholipids in vesicle systems. (A) Schematic of DOPA synthesis from free fatty acids. Oleic acid, ATP, and glycerol 3-phosphate are utilized in a three-step reaction (catalyzed by FadD, PlsB, and PlsC) to produce the phospholipid DOPA. CoA is required as a cofactor. (B) Chemical structures of oleic acid and DOPA. (C) Schematic of the two-compartment vesicle system: vesicles equipped with the l-arginine breakdown pathway and machinery for glycerol 3-phosphate synthesis and export (feeder, purple) are separated from vesicles for DOPA synthesis (utilizer, green) by a polycarbonate dialysis filter with 50 nm pore size, which is permeable for small molecules. (D) Molecular composition of feeder and utilizer vesicles. ArcD from *L. sakei* was used in this experimental setup. (E) Synchronous ATP recycling and glycerol 3-phosphate formation in the feeder vesicles as shown by the time course of the ATP/ADP ratio (representative traces from 3 independent reconstitutions). ATP and glycerol 3-phosphate formation was started at *t* = 0 by addition of 20 mM l-arginine plus 0.4 mM glycerol. (F) Feeder-dependent DOPA biosynthesis by utilizer vesicles detected by LC-MS. Mg-ATP (2 mM) was added at *t* = 0 (*n* = 3 independent reconstitutions; error bars are s.e.m.).

Next, we couple the module for ATP recycling and building block formation to one that enables the synthesis of phospholipids. We develop the modules in distinct vesicles for better control of the reaction networks. We used the *E. coli* acyltransferases PlsB and PlsC in combination with FadD, a soluble enzyme that catalyzes acyl-CoA formation from free fatty acids in the presence of ATP and CoA (Figure 5a,b);^13^ the lipid-synthesizing enzymes of the utilizer vesicles have a topology similar to that in the endoplasmic reticulum of eukaryotic cells. We achieve synthesis of the lipid DOPA by feeding glycerol 3-phosphate from the feeder to utilizer vesicles that consume glycerol 3-phosphate to produce lipids.

To prevent possible solubilizing effects of the lipid precursors, we spatially segregated l-arginine-driven glycerol 3-phosphate formation and export (the “feeder” module) from DOPA biosynthesis (the “utilizer” module) by separating the two types of vesicles with a polycarbonate dialysis filter of pore size of ∼50 nm (Figure 5c,d). We exploited our previously developed multichamber dynamic dialysis setup^10^ but did not use the continuous flow needed for dynamic studies. Importantly, small water-soluble metabolites, such as glycerol 3-phosphate, diffuse along their concentration gradient from the feeder to the utilizer module.^10^ Long-chain fatty acids do not cross the dialysis membrane due to their self-assembly into large supramolecular structures in a pH-dependent manner (i.e., vesicles at 7 < pH < 8) (Figure S8).

### Coupling of Modules for Lipid Biosynthesis

We filled one compartment of the dialysis chamber with feeder vesicles containing both the l-arginine breakdown pathway and the glycerol 3-phosphate synthesis module. We added 5 mM fructose 1,6-bisphosphate (FBP) to inhibit external GlpK, in case some enzyme would leak from the vesicles.^27^ (Figure 5d, upper panel). ATP from the l-arginine breakdown pathway is used to synthesize glycerol 3-phosphate in the presence of 0.4 mM glycerol. Glycerol 3-phosphate is subsequently exported out of the feeder vesicles and it diffuses to the compartment with the utilizer vesicles. This compartment was filled with vesicles decorated with FadD, PlsB, and PlsC and was supplemented with 400 μM oleic acid, 2 mM Mg-ATP, and 0.1 mM CoA (Figure 5d, lower panel).

Vesicles with and without GlpT synthesized glycerol 3-phosphate, as indirectly shown by the ATP/ADP ratio recorded in parallel experiments (Figure S7). Full ATP/ADP recovery after 1.5 h was observed for vesicles carrying the glycerol 3-phosphate/Pi antiporter, indicating that all glycerol had been converted into glycerol 3-phosphate (Figure 5e). In agreement, the control vesicles without GlpT failed to recover their ATP/ADP levels because the internal Pi was depleted. The minor ATP/ADP increase observed upon substrate addition to GlpT-deficient vesicles suggests that a subpopulation of vesicles lacks GlpK. This is in line with previous observations that a fraction of the vesicles lacks one or more biomolecules due to the stochastic distribution of the components in the encapsulation process.^10^

Phospholipid biosynthesis was measured by LC-MS analysis of samples collected over time from the compartment with the utilizer vesicles. Little or no DOPA synthesis was observed in the first 2 h, which is the time frame required for the diffusion of glycerol 3-phosphate from the feeder (synthesis and export) to the utilizer compartment (Figure 5f). DOPA was then formed but only when the feeder vesicles were equipped with the glycerol 3-phosphate exporter GlpT; the control without GlpT did not show any DOPA formation. As two fatty acid molecules are needed to form one phospholipid, a maximum yield of 200 μM DOPA would be possible if all of the molecules would be available for synthesis. However, at pH 7, most of the fatty acids are present in the form of vesicles from where the molecules are slowly released. After 6 h, 5.7 μM DOPA was produced. The diffusion of glycerol 3-phosphate from one compartment to the other is limiting the feeding of building blocks between different vesicles (Figure S8).

## Discussion

Robust and sustainable energy recycling is essential for autonomously functioning metabolic networks, ultimately leading to the design of synthetic cells.^28^ Here, we present an encapsulated l-arginine-dependent ATP recycling pathway that utilizes a novel l-arginine/l-ornithine antiporter with enhanced transport capacity. We equipped the vesicles with a module for glycerol 3-phosphate synthesis and coupled its export to the import of inorganic phosphate, thereby avoiding internal depletion of free phosphate. This yielded a fully sustainable synthetic metabolic network where all nutrients are imported, cofactors recycled, and end products exported, which is now only limited by the availability of external substrates. Hence, a true out-of-equilibrium, or selectively open, metabolic network, akin to how living cells work, but at the same time it is unique due to its bottom-up construction from components of different (biological) origin.

The performance of ATP-generating pathways is often qualitative, and it is not clear whether existing systems would be able to sustain the global energetic needs of an autonomous synthetic cell. From our data, we calculated that our ATP-producing module would allow a cell to grow with a doubling time of 10 days. While this appears much slower than that of model organisms grown under optimal laboratory conditions, in nature, many microorganisms grow with comparable doubling times, often because they are limited by nutrient availability. In addition, we foresee that the reconstitution of complex processes (e.g., DNA duplication and segregation, protein synthesis, cell division, etc.) in autonomous synthetic cells may initially not require much faster synthesis of ATP, so l-arginine breakdown could be exploited as a simple yet effective module for sustainable energy generation. Net import or *de novo* synthesis of adenine nucleotides will additionally be required when ATP is consumed as a building block (e.g., for nucleic acid synthesis).

To showcase the potential of continuous and sustainable ATP recycling, we coupled l-arginine-breakdown-driven glycerol 3-phosphate synthesis to another essential metabolic process, i.e., lipid synthesis. Low phospholipid yields have been previously reported for *in vitro* transcription-translation-based systems reconstituted in giant vesicles due to the limited solubility of the acyl-chain precursors and impaired ribosomal processivity.^16,29,30^ In our system, phospholipid formation is limited by the equilibration of glycerol 3-phosphate through the dialysis filter; higher yields are expected in bulk,^13^ provided that the lipid-producing vesicles can be stabilized against leakage (e.g., with a peptide or PEG coating).

The bottom-up construction of complex metabolic systems inspired by eukaryotic cells is gaining momentum.^31^ For instance, a multistep enzymatic cascade has been engineered in vesicles, where each reaction step is spatially segregated into a subcompartment and metabolic intermediates diffuse through membrane pores.^32^ Living cells have also been exploited as organelle components of metabolic synthetic cells.^33^ Artificial β-cells have been engineered that sense high glucose levels *in vitro* and *in vivo*, and they respond by releasing insulin from internal storage vesicles, effectively restoring physiological glucose concentrations.^34^

The vesicle systems presented in this work could be developed toward ATP-producing (“the feeder vesicles”) and lipid-synthesizing (“the utilizer vesicles”) organelles or used for coupling to other metabolic pathways and studying their behavior and emerging properties under defined conditions. Such coupled reaction networks may also find application as functional containers for the construction of autonomously growing synthetic cells of a larger diameter (ideally, bacteria-sized giant unilamellar vesicles^28^). We argue that multicompartment synthetic cells have a more favorable surface-to-volume ratio (a parameter important for metabolite exchange and other membrane-related processes^35^) than cells that lack organelles, but autonomous growth of such cells also requires an approach for the duplication of the added compartments. The spatial confinement of metabolic modules into different subcompartments provides a higher control over the construction of complex reaction networks, as each organelle is relatively simple (e.g., in terms of the number of components). This will minimize stochastic effects and facilitate modeling the processes.^28^ Finally, the physical separation of reaction networks into synthetic organelles will allow the coexistence of mutually exclusive processes that would otherwise be impaired in a single compartment.

In conclusion, we have coupled two essential metabolic processes, i.e., ATP recycling and lipid synthesis, in a two-vesicle system that is highly tunable and can be expanded in a modular fashion or combined with other independently designed reaction networks. We exploit the use of substrate/product antiporters to maintain out-of-equilibrium reaction conditions by coupling the uptake of the substrate to the secretion of metabolic products.

## Methods

### Chemicals and Media

All chemicals and media were as reported previously,^10^ with the following additions: 1,4-dithiothreitol (Carl Roth); ampicillin sodium salt (Formedium); ^14^C-l-arginine (stock solution 325 mCi/mmol; NovaTec); 2-mercaptoethanol (Sigma-Aldrich); chloramphenicol (Carl Roth); coenzyme A, free acid (Avanti Polar Lipids); d-(+)-fructose 1,6-bisphosphate trisodium salt hydrate (Sigma-Aldrich); d(+)-glucose anhydrous (Formedium); d(+)-sucrose (Formedium); ethylenediaminetetraacetic acid disodium salt dihydrate (Sigma-Aldrich); HEPES (Carl Roth); methanol (Sigma); *n*-butanol (Sigma); *n*-dodecyl-β-d-maltopyranoside, Anagrade (Anatrace); oleic acid (Sigma-Aldrich); tris hydrochloride (ITW Reagents); and TritonTM X-100 (Sigma-Aldrich).

### Strains and Plasmids

Genomic DNA from *C. autoethanogenum* 9 (DSM 10061), *P. aeruginosa* PAO1 (DSM 22644), and *Roseobacter denitrificans* ATCC 33942 (DSM 7001) were purchased from DSMZ. Genomes from *Lactobacillus fermentum* IMDO 130101 and *L. sakei* ATCC 15521 were kindly donated by Professor Frederic Leroy and Professor Jan Kok, respectively. A codon-optimized synthetic gene was ordered for ArcD from *Rhizobium fredii* HH103 (GenScript). Template vectors encoding *Lactobacillus brevis* ATCC 367 *arcD* and *S. pneumoniae* D39 *arcE* were generously provided by Dr. Juke S. Lolkema. Expression vectors encoding *arcA*, *arcB*, *arcC1* and *arcD2*,^9^*percevalHR*, and *glpK*,^10^ as well as *fadD*, *plsB*, and *plsC*(13) were as previously reported; a plasmid carrying *glpT*(25) was a kind gift of Professor Da-Neng Wang (Table S2).

### Cloning of Genes for ArcD and ArcE Homologues

The genes encoding *arcD/E* were PCR-amplified from the source DNA templates with Phusion high-fidelity DNA polymerase (Thermo Fisher Scientific) and customized primers (Eurofins Genomics, Table S3). Next, fragment exchange (FX)^36^ was exploited to clone the genes of interest into pNZ-N(His) or pNZ-C(His) vectors suitable for overexpression in *L. lactis* strains. According to the procedure, the PCR amplification products were first cloned into a sequencing vector (pINITIAL). To this end, both the insets and the backbone (1:5 molar ratio) were digested with 2.5 U of *Sap*I (New England Biolabs) for 1 h at 37 °C, followed by a heat inactivation step at 60 °C for 20 min. Ligation was carried out by adding 5 U of T4 ligase (New England Biolabs) and incubating for 1 h at RT. The plasmids were transformed by heat shock into chemically competent *E. coli* MC1061 cells that were then plated onto lysogeny broth (LB) supplemented with 1% w/w agar and 5 μg/mL chloramphenicol. After overnight incubation at 37 °C, single colonies were used to prepare 2 mL cultures that were grown overnight at 37 °C and 200 rpm and used for plasmid isolation, followed by DNA sequencing (Eurofins Genomics, Figure S1). The genes of interest were then transferred from pINITIAL*arcD/E* into the intermediate pREX (1:4 molar ratio) by following the same procedure described above, with the difference that LB agar plates containing 7% w/w sucrose and 100 μg/mL ampicillin were used for selection. Finally, *arcD/E* genes were cloned into the expression plasmids pNZ-N(His) or pNZ-C(His) by vector backbone exchange (VBEx).^36^ Briefly, pREX*arcD/E* and the acceptor vector pERL were mixed (1:3 molar ratio) and digested by incubating with 3 U of *Sfi*I (New England Biolabs) for 2 h at 50 °C, followed by enzyme inactivation and ligation for 2 h at RT. The obtained constructs were transformed by electroporation into electrocompetent *L. lactis* JP9000 Δ*arcD1*Δ*arcD2* cells^24^ that were then plated onto M17 medium supplemented with 1% agar, 1% w/w glucose, and 5 μg/mL chloramphenicol (GM17Cm-agar) and incubated o/n at 30 °C.

### Overexpression and Purification of ArcD Homologues

Large-scale cultures were prepared for *L. lactis* JP9000 Δ*arcD1*Δ*arcD2* carrying pNZ*arcD2*Δ*C* or pNZ*arcD-LS* in a 10 L bioreactor, followed by cell lysis and membrane vesicle isolation according to a standardized procedure.^9^ Next, *L. lactis* ArcD2 and *L. sakei* ArcD were purified by affinity chromatography as described previously,^8−10^ with the important difference that 2 mM β-mercaptoethanol plus 10% v/v glycerol were added during membrane vesicle solubilization and maintained in the buffer (50 mM KPi, 200 mM KCl, 0.5% w/v DDM, 2 mM β-mercaptoethanol, and 10% v/v glycerol) throughout the purification procedure. Elution was performed without glycerol for immediate reconstitution in LUVs (see below), while 10% v/v glycerol was added for the storage of the proteins at −80 °C.

### Overexpression and Purification of GlpT

#### Overexpression

Chemically competent *E. coli* MC1061 cells were transformed with pBADmycHisB-*glpT* by heat shock, plated onto LB agar plates supplemented with 100 μg/mL ampicillin, and incubated overnight at 37 °C. A preculture was prepared by inoculating a fresh colony in 175 mL LB-amp, followed by incubation overnight at 37 °C, 200 rpm. The o/n precultures were diluted 1:40 in 6× 1 L fresh LB-amp and grown at 37 °C until an OD_600_ of 0.5 was reached. The temperature was lowered to 25 °C, and overexpression was induced with 0.01% l-arabinose at an OD_600_ of 0.94. After 2 h of expression, cells were harvested by centrifugation (15 min, 6000*g*, 4 °C), washed with 50 mM Tris-HCl pH 8.0, and resuspended to a final concentration of 220 g/L with the same buffer supplemented with 20% w/w glycerol. Cells were flash-frozen with liquid N_2_ and stored at −80 °C for later use.

#### Membrane Vesicle Preparation

Membrane vesicles were prepared by thawing 11 g of cells and diluting them to 110 g/L in 100 mL of buffer of the final composition 50 mM Tris-HCl pH 8.0 plus 400 mM NaCl, to which were added 1 mM PMSF, 1 mM MgCl_2_, and 100 μg/mL DNase. Cells were disrupted by a single passage through an HPL6 press (Maximator GmbH) at 20 KPsi, 4 °C and supplemented with 5 mM Na-EDTA pH 8.0. Membrane vesicles were purified from the cellular debris by high-speed centrifugation (15 min, 15.000*g*, 4 °C) and pelleted by ultracentrifugation (45 min, 175.000*g*, 4 °C). The pellets were washed with 25 mL 50 mM Tris-HCl pH 8.0 and the ultracentrifugation step was repeated. Membrane vesicles were resuspended to a final concentration of 21 mg/mL in 10 mL of 50 mM Tris-HCl pH 8.0 plus 20% w/w glycerol, flash-frozen with liquid N_2_ and stored at −80 °C for later use.

#### Purification

A membrane vesicle aliquot containing 10 mg of total membrane proteins was solubilized with 0.5% w/w DDM in solubilization buffer (50 mM Tris-HCl pH 8.0, 20% w/w glycerol, 10 mM imidazole) in a 30 min nutation step at 4 °C. The debris was removed by ultracentrifugation (12 min, 328.000*g*, 4 °C), and the solubilized protein was loaded onto 0.25 mL (1 CV) of Ni-Sepharose prewashed with an excess of Milli-Q water. Binding was performed for 1 h at 4 °C, with nutation. The flow-through was removed, and the protein was washed (20 CVs, 50 mM Tris-HCl pH 8.0, 20% w/w glycerol, 0.04% w/w DDM, 50 mM imidazole) and eluted by increasing the imidazole concentration in the washing buffer to 500 mM. The peak fractions from the Ni-Sepharose were pooled and then loaded onto a Superdex 200 (GE Healthcare) column, equilibrated in 50 mM HEPES, pH 7.0 plus 150 mM NaCl and 20% w/w glycerol. The elution fractions were concentrated to 1.2 mg/mL with an Amicon Ultra-0.5 centrifugal filter unit (Merck) with a cutoff of 100 kDa, flash-frozen with liquid N_2_ and conserved at −80 °C.

### Overexpression and Purification of Other Proteins

ArcA, ArcB, ArcC1, PercevalHR and GlpK, and FadD, PlsB, and PlsC were overexpressed and purified as previously reported.^8−10,13^

### Formation of Proteoliposomes with One or Multiple Antiporters

Purified *L. lactis* ArcD2 and *L. sakei* ArcD were reconstituted separately in liposomes composed of DOPE/DOPG/DOPC at 25:25:50 mol % with a lipid-to-protein ratio of 400:1 w/w. The Triton X-100-mediated vesicle destabilization protocol^8−10^ was adapted by adding 2 mM DTT to the reconstitution buffer. To equip vesicles with *E. coli* GlpT, the antiporter was coreconstituted together with the l-arginine/l-ornithine antiporter at a lipid-to-protein ratio of 400:1 w/w. The overall lipid-to-protein ratio was 200:1 w/w.

### *In Vitro*l-Arginine Uptake

l-Arginine/l-ornithine exchange activity was determined via the uptake of radiolabeled l-arginine in vesicles containing *L. lactis* ArcD2 or *L. sakei* ArcD at a lipid-to-protein ratio of 400:1 w/w. l-Ornithine was encapsulated at a concentration of 10 mM (10 mg, 400 μL) resuspended in 50 mM KPi pH 7.0 plus 2 mM DTT by 5 freeze–thaw cycles. The thawing step was performed in an ice–water bath at 10 °C. Proteo-LUVs were extruded 13× through a 400 nm polycarbonate filter pre-equilibrated with 10 mM l-ornithine, 2 mM DTT, 50 mM KPi pH 7.0, diluted to 6 mL with 50 mM KPi pH 7.0, 2 mM DTT without l-ornithine and collected by centrifugation (20 min, 325,000*g*, 4 °C). Remaining external l-ornithine was diluted further by an additional resuspension of the pellet in 6 mL of 50 mM KPi pH 7.0 plus 2 mM DTT and centrifugation (20 min, 325,000*g*, 4 °C). Collected proteo-LUVs containing l-ornithine were finally resuspended to a lipid concentration of 200 mg/mL in 50 mM KPi pH 7.0 plus 2 mM DTT.

For the uptake assays, encapsulated proteo-LUVs were diluted 50 times in the preheated (30 °C) reaction buffer containing 25 μM ^14^C-l-arginine, 2 mM DTT, and 50 mM KPi pH 7.0. A stock of ^14^C-l-arginine with a specific activity of 325 mCi/mmol was diluted 125 times with unlabeled l-arginine. A volume of 100 μL was taken from the reaction mixture at 0, 5, 10, 25, 40, and 60 s, diluted into 2 mL of ice-cold 50 mM KPi pH 7.0, and filtered through a 0.45 μm cellulose nitrate filter. The filter was further washed with 2 mL of ice-cold 50 mM KPi at pH 7.0 and dissolved in Ultima Gold MV scintillation liquid (PerkinElmer). Radioactivity was quantified in a Tri-Carb 2800TR scintillation counter (PerkinElmer). For the determination of kinetic parameters of *L. sakei* ArcD, external l-arginine in the reaction solution was varied between 1–100 μM. Initial rates of l-arginine uptake were plotted versus the external l-arginine concentration and fitted to a Michaelis–Menten equation to obtain the *K*_M_^arg^ and *V*_max_ values.

### Encapsulation of Soluble Components for l-Arginine Breakdown and Glycerol 3-Phosphate Formation

A previously described procedure^10^ was followed for the encapsulation in vesicles of the l-arginine breakdown enzymes, glycerol kinase, fluorescent sensors, and small molecules, with minor adaptations. Briefly, 10 mM Na-ADP, 10 mM MgCl_2_, 0.5 mM l-ornithine, 37.5 μg of ArcA, 96 μg of ArcB, 72 μg of ArcC1, and 71 μg of PercevalHR plus 35.8 μg of GlpK were mixed in 50 mM KPi pH 7.0 plus 2 mM DTT to a final volume of 200 μL. This mixture was added to preformed proteoliposomes bearing either *L. lactis* ArcD2 or *L. sakei* ArcD and *E. coli* GlpT; control vesicles were prepared without GlpT. For experiments that did not require glycerol 3-phosphate formation, GlpK was omitted from the mixture and ArcD-only proteoliposomes were used. Five freeze–thaw cycles were performed, followed by a 13× extrusion step through a 400 nm polycarbonate filter (Whatman, GE Healthcare). Extruded vesicles were thoroughly washed with 3 × 6 mL of the external buffer (50 mM KPi pH 7.0, 58 mM NaCl, 2 mM DTT) by centrifugation (30 min, 325,000*g*, 4 °C). The vesicles were resuspended to a stock concentration of 5.55 mg of total lipids/mL and used immediately or stored at 4 °C up to 48 h.

### ATP Quantification by Chemiluminescence

Chemiluminescence experiments were carried out similarly as previously reported.^10^ Briefly, vesicles equipped with either ArcD2 from *L. lactis* or ArcD from *L. sakei*, plus the soluble components required for l-arginine breakdown, were diluted to a concentration of 2.7 mg of total lipids/mL with the external buffer (50 mM KPi pH 7.0, 58 mM NaCl, 2 mM DTT) and mixed with 1 μM valinomycin plus 1 μM nigericin. A 100 μL sample was taken at *t* = 0, followed by incubation at 30 °C and addition of 5 mM l-arginine to initiate ATP formation. Samples were collected over a time course of 2 h, quenched by perchloric acid treatment, and analyzed as described before.^10^

### ATP/ADP Determination by Fluorescence

ATP/ADP measurements were carried out in parallel with ATP quantification as previously reported.^10^ Briefly, a volume of 120 μL containing 2.7 mg of total lipids/mL vesicles (in 50 mM KPi pH 7.0, 58 mM NaCl, 2 mM DTT) equipped with the l-arginine breakdown pathway were mixed with ionophores (1 μM valinomycin plus 1 μM nigericin) in ultra-microcuvettes 105.252-QS (Hellma Analytics) and incubated at 30 °C in a FP-8300 or FP-8350 fluorimeter (Jasco). ATP formation was initiated by the addition of 5 mM l-arginine, and PercevalHR excitation spectra were acquired over time (excitation = 400–520 nm, bandwidth = 5 nm; emission = 550 nm, bandwidth = 5 nm). The ATP/ADP recovery experiments were carried out analogously but with vesicles that carried GlpT and GlpK in addition to the components for the l-arginine breakdown pathway. The reaction was started by the addition of 5 mM l-arginine and incubated until a plateau was reached (∼60 min for *L. lactis* ArcD2 and ∼30 min for *L. sakei* ArcD). Next, glycerol was added at different concentrations (0–700 μM), together with 5 mM fresh l-arginine (5 mM). Additions of 5 mM l-arginine were made at 3–4 h intervals. PercevalHR excitation spectra were recorded as described.

### DOPA Biosynthesis in Feeder–Utilizer Vesicles

#### Feeder Proteo-LUVs

Vesicles equipped with l-arginine breakdown (ArcD *L. sakei*) and glycerol 3-phosphate synthesis plus export were used at a final concentration of 2.7 mg of total lipids/mL in 50 mM KPi pH 7.0, 58 mM NaCl, and 2 mM DTT. A 0.8 mL reaction mixture was prepared by adding 1 μM valinomycin, 1 μM nigericin, and 5 mM fructose 1,6-bisphosphate (FBP). Control samples were prepared in the same way but with vesicles without GlpT. The vesicles were kept at 4 °C until the reaction was started.

#### Utilizer Vesicles

Empty phospholipid vesicles composed of DOPE/DOPG/DOPC at 25:25:50 mol % were prepared with 50 mM KPi, 58 mM NaCl plus 2 mM DTT as the internal and external buffer. The empty vesicles were used at a final concentration of 2.7 mg of total lipids/mL (3.5 mM) in a 0.8 mL reaction volume. 0.4 mM oleic acid, 0.1 mM CoA, 6.2 μg (0.2 μM) of FadD, 9.2 μg (0.2 μM) of PlsB, and 2.8 μg (0.2 μM) of PlsC were added to the sample. The vesicles were kept at 4 °C until the reaction was started.

#### Dialysis Setup and Procedure

Four chambers of a previously described dynamic dialysis setup were equipped with a 50 nm polycarbonate filter (Avestin) according to the instructions.^10^ Next, the flow inlet and outlet tubes of each chamber were tightly sealed to create a two-compartment dialysis setup without flow. The four dialysis chambers were connected to a water flow set to 30 °C, filled with buffer (50 mM KPi pH 7.0, 58 mM NaCl, 2 mM DTT), and allowed to pre-equilibrate overnight with stirring. The following day, the pre-equilibration buffer was removed, and the feeder and utilizer vesicles were transferred to their respective compartments in the dialysis chambers, each with gentle stirring. An 80 μL time point was collected for LC-MS analysis from the utilizer vesicle compartment prior to the addition of substrates. Then, ATP recycling and glycerol 3-phosphate formation and export were initiated by the addition of 20 mM l-arginine plus 0.4 mM glycerol to the feeder vesicles, while DOPA biosynthesis was started by the addition of 2 mM Mg-ATP to the utilizer vesicles. Additional samples were taken over a time course of 8 h; all samples were immediately quenched by adding 300 μL of *n*-butanol and 5 mM Na-EDTA and vortexing thoroughly. The activity of the feeder vesicles in the presence of 5 mM FBP and upon concomitant addition of 20 mM l-arginine plus 0.4 mM glycerol was followed in parallel by determining the ATP/ADP levels by fluorescence.

### LC-MS Analysis of Phospholipids

#### Lipid Extraction

The *n*-butanol-quenched reaction samples (total volume 380 μL) were centrifuged at 10.000 rpm and RT for 2 min. The lipid-containing organic phase (top layer) was collected and transferred to clean 1.5 mL screw neck vials (VWR), and the extraction process was repeated with 300 μL of fresh *n*-butanol, followed by *n*-butanol evaporation under a gaseous nitrogen flow. The lipid film was then resuspended in 50 μL of methanol, transferred into a 0.1 mL micro insert (VWR), and analyzed by LC-MS.

#### LC-MS Analysis

Extracted lipids were analyzed using an established protocol^37^ using an Accela1250 high-performance liquid chromatography (UHPLC) system coupled to a heated electrospray ionization–mass spectrometry (HESI-MS) Orbitrap Exactive (Thermo Fisher Scientific) mass spectrometer. A sample of 5 μL was injected into an ACQUITY UPLC CSH C18 1.7 μm column, 2.1 mm × 150 mm (Waters Chromatography Ireland Ltd.) operating at 55 °C with a flow rate of 300 μL/min. Separation of the compounds was achieved by a changing gradient of eluent A (5 mM ammonium formate in water/acetonitrile 40:60, v/v) and eluent B (5 mM ammonium formate in acetonitrile/1-butanol, 10:90, v/v), with the exception of the data displayed in Figure S8, in which 1-butanol of eluent B was replaced by 2-propanol. The following linear gradient was applied: 45% eluent B for 2.5 min; a gradient from 45 to 90% eluent B over 19.5 min; holding for 3 min; returning to 45% eluent B in 0.5 min; and holding for 8 min. The column effluent was injected directly into the Exactive ESI-MS Orbitrap mass spectrometer operating in negative ion mode. Voltage parameters of 3 kV (spray), −75 V (capillary), −190 V (tube lens), and −46 V (skimmer voltage) were used. A capillary temperature of 300 °C, a sheath gas flow of 60, and an auxiliary gas flow of 5 were maintained during the analysis. Spectral data constituting total ion counts were analyzed using Thermo Scientific XCalibur processing software (Table S6 and Figure S9).

## Data Availability

All data are available in the main manuscript and the Supporting Information; raw data are available upon request.

## Supporting Information Available

Amino acid sequence alignment and identity of ArcD and ArcE proteins; predicted topology of ArcD and ArcE proteins; nucleotide sequence of *arcD and arcE* genes; *in vivo*l-arginine uptake by ArcD and ArcE transport proteins; SDS-PAA gel images of purified membrane proteins used in this study; l-arginine-mediated ATP formation in the presence of *L. sakei* ArcD or *L. lactis* ArcD2; activity of the feeder LUVs measured by online ATP/ADP readout with PercevalHR; diffusion of phospholipid precursors through polycarbonate filters; the calibration curve for DOPA quantification; an overview of candidate ArcD/E homologues; plasmids used in this study; primers used in this study; kinetic parameters of ArcD and ArcE proteins based on *in vivo*l-arginine uptake; kinetic parameters of *L. sakei* ArcD and *E. coli* GlpT; and the mass-to-charge ratio of lipid species (PDF)

## Abbreviations

ADP: adenosine diphosphate
ATP: adenosine triphosphate
CL: cardiolipin
DDM: *n*-dodecyl-β-maltoside
DOPA: dioleoyl phosphatidic acid
DOPE: dioleoylphosphatidyl ethanolamine
DOPG: dioleoylphosphatidyl glycerol
DTT: dithiothreitol
EDTA: ethylenediaminetetraacetic acid
FBP: fructose 1,6-bisphosphate
LC-MS: liquid chromatography–mass spectrometry
LUV: large unilamellar vesicle
MFS: major facilitator superfamily
PA: phosphatidic acid
PCA: perchloric acid
PE: phosphatidyl ethanolamine
PG: phosphatidyl glycerol
PMF: proton motive force
