# Supplementary material for: Synthetic Vesicles for Sustainable Energy Recycling and Delivery of Building Blocks for Lipid Biosynthesis†
Source: ACS Synth Biol. 2024 Apr 18;13(5):1549–61. doi: 10.1021/acssynbio.4c00073 (PMC11106768; doi:10.1021/acssynbio.4c00073)
Supplement: Supplementary file 1 — sb4c00073_si_001.pdf [file sb4c00073_si_001.pdf]

Supporting Information

Synthetic vesicles for sustainable energy recycling and delivery of building blocks for lipid biosynthesis

Eleonora Bailoni[1], Miyer F. Patiño-Ruiz[1], Andreea R. Stan[1†], Gea K. Schuurman-Wolters[1], Marten Exterkate[2], Arnold J.M. Driessen[4] and Bert Poolman[1*]

[1]Department of Biochemistry, and [4]Department of Molecular Microbiology
Groningen Biomolecular Sciences and Biotechnology Institute Nijenborgh 4, 9747 AG, Groningen, The Netherlands

[2]Department of Membrane Biogenesis and Lipidomics, Institute of Biochemistry, Heinrich-Heine-Universität Düsseldorf, Universitätsstraβe 1, 40225, Düsseldorf, Germany

*To whom correspondence should be addressed (email: b.poolman@rug.nl)

[†]The author deceased before publication

Keywords: ATP recycling | glycerol 3-P/Pi antiporter | out-of-equilibrium metabolic network | phospholipid biosynthesis | building block delivery | synthetic cells

Supplementary Figures and Tables:

Figure S1. **Amino acid sequence alignment and identity of ArcD and ArcE proteins.**

Figure S2. **Predicted topology of ArcD and ArcE proteins.**

Figure S3. **Nucleotide sequence of *arcD and arcE* genes.**

Figure S4. ***In vivo* L-arginine uptake by ArcD and ArcE transport proteins.**

Figure S5. **SDS-PAA gel images of purified membrane proteins used in this study.**

Figure S6. **L-arginine-mediated ATP formation in the presence of *L. sakei* ArcD or *L. lactis* ArcD2.**

Figure S7. **Activity of the feeder LUVs measured by online ATP/ADP readout with PercevalHR.**

Figure S8. **Diffusion of phospholipid precursors through polycarbonate dialysis filters.**

Figure S9. **Calibration curve for DOPA quantification.**

Table S1. **Overview of candidate ArcD/E homologues.**

Table S2. **Plasmids used in this study.**

Table S3. **Primers used in this study.**

Table S4. **Kinetic parameters of ArcD and ArcE proteins based on *in vivo* L-arginine uptake.**

Table S5. **Kinetic parameters of L. sakei ArcD and E. coli GlpT.**

Table S6. **Mass-to-charge ratio of lipid species used in this study.**

Table S1. Overview of ArcD and ArcE homologues. The *L. lactis* IL 1403 ArcD2[1] served as a reference for the screening of homologues by genome database searches.

Protein	Organism	ID	MW (KDa)	Sequence identity (%)	Predicted number of TM α–helices
ArcD	*Clostridium autoethanogenum* 9	A0A3M0SNP9	50.8	38.44	13
	Lactobacillus brevis ATCC 367	Q03NZ0	50.5	44.64	13
	Lactobacillus fermentum IMDO 130101	B7VBS0	51.1	39.79	13
	Lactobacillus sakei ATCC 15521	G3ADI7	51.8	45.18	13
	Lactococcus lactis IL1403	Q9CE19	54.0	100	13
	Pseudomonas aeruginosa PAO1	P18275	52.0	36.21	13
	Rhizobium fredii HH103	G9A0G0	51.0	30.45	13
	Roseobacter denitrificans ATCC 33942	Q16C52	46.3	31.10	11
ArcE	*Lactobacillus brevis* ATCC 367	Q03NY7	56.5	21.73	13
	Lactobacillus sakei ATCC 15521	G3ADI9	56.1	19.87	12
	Streptococcus pneumoniae D39	A0A0H2ZNX2	54.0	18.32	13

Figure S1. Amino acid sequence alignment and identity of ArcD and ArcE proteins. The amino acid sequence alignments and percent identity were obtained with Clustal Omega[2]. *L. lactis* ArcD2 and *L. sakei* ArcD sequences are 45% identical. *L. lactis* ArcD2 and the ArcE homologues have a low sequence identity (<30%).

ArcD homologues, amino acid sequence alignment

```
tr|A0A3M0SNP9|A0A3M0SNP9_9CLOT      ---MSENNKLGLFSLIALVIGSMIGGGAFSLPGDMAKGASAGAIIIGWLITGIGMIALAF   57
tr|Q16C52|Q16C52_ROSDO              ----------------------------------MD-DCRNRNVHAGACVSV--------   17
sp|P18275|ARCD_PSEAE                -MSQESSQKLRLGALTALVVGSMIGGGIFSLPQNMAASADVGAVLIGWAITAVGMLTLAF   59
tr|G9A0G0|G9A0G0_RHIFH              --MTSTAQKLSLASLSALVVGSMVGAGIFSLPRTFGNATGPFGAIVAWCIAGAGMFTLAH   58
tr|Q9CE19|Q9CE19_LACLA              -MENKKTKGISLFALLAIIISGAIGGGVFNLANDLANGSTPGGVMISWLFIGFGIFMLVL   59
tr|B7VBS0|B7VBS0_LACFE              ---MENKNGIGRIGLIALIVSSCIGTGIFGITNAVAAAAAPGPALLAWLFVGFGFLMLVF   57
tr|G3ADI7|G3ADI7_LACSK              MTEEKPAKKIGLLALIALVISSSIGSGVFGLTSDLASASAPGPVLIAWVIVGFGILMLAL   60
tr|Q03NZ0|Q03NZ0_LACBA              ---MDENKGLSMGALTAAVVTSSIGSGVFTLTSSLAGGAAAGPVLLAWLVVGFGILMLAL   57
                                                                      .           .  .

tr|A0A3M0SNP9|A0A3M0SNP9_9CLOT      VYQNLSMKRPDLNGGIYSYAKAGFGGYMGFNSAWGYWLSALIGNVSYLVMMFGAVGYFFP   117
tr|Q16C52|Q16C52_ROSDO              ----SGAATSQSGFGRFCYAKAGFGSYVGFLSASGYGASAMLGNVFYWVLISATLSLFFP   73
sp|P18275|ARCD_PSEAE                VFQTLANRKPELDGGVYAYAKAGFGDYMGFSSAWGYWISAWLGNVGYFVLLFSTLGYFFP   119
tr|G9A0G0|G9A0G0_RHIFH              VFRVLAERKPDLDAGVYAYAKAGFGDYAGFLSALGYWLVGCIADVSYWVLIKATLGAFFP   118
tr|Q9CE19|Q9CE19_LACLA              SFNRLITIRPKLS-GVSDYAREGFGDFIGFLSGWGYWISAWTGTIGFAVLMMTSADYFFP   118
tr|B7VBS0|B7VBS0_LACFE              ALNNLSEKRPDLEAGIFSYAGAGFGPMGEFISGWAYWLSAWLGNIAFATMLMSALGTFFP   117
tr|G3ADI7|G3ADI7_LACSK              SLNNLLMKEPELE-GIFSYAEKGFGPFAGFISGWGYWLSAWLGNVAFATILMSALGYFFP   119
tr|Q03NZ0|Q03NZ0_LACBA              SLNNLLQKNPEAE-GVQAYAQAGFGNFAGFVSGWGYWLSAWLGNVAFATVLMSSLGYFFP   116
                                              .   *   **  ***    * *. .*   .  . : : .::  : . ***

tr|A0A3M0SNP9|A0A3M0SNP9_9CLOT      VFGK----GNNLASVVCASIMLWLIQGLILKGVKQAAIVNVITTIAKLVPIFLFVIIAII   173
tr|Q16C52|Q16C52_ROSDO              AFGD----GGTILGIVVSLIGIWTFHFIILRGIQEAAFINTVVSIAKLVPLFVAITAMVF   129
sp|P18275|ARCD_PSEAE                IFGK----GDTVAAIVCASVLLWALHFLVLRGIKEAAFINTVTTVAKVVPLFLFILICLF   175
tr|G9A0G0|G9A0G0_RHIFH              IFGD----GNTVAAVLVSSVALWGFHFMILRGIKEAAAINTVVTVAKIVPIVIFIVILLG   174
tr|Q9CE19|Q9CE19_LACLA              SKFANSNGSLTVLSVIIVSIISWILMFLVDRGVETAALVNAVVMIAKLIPLVVFSITGII   178
tr|B7VBS0|B7VBS0_LACFE              VFGN----GQNLTSIIVAIIFCWVLTLLVNSGVETATFINTIGTFFKIIPLVLFIIISII   173
tr|G3ADI7|G3ADI7_LACSK              IFKS----GQNLPSILVASVLSWSLTYFVNRGVEGAAAINTLVTICKLIPLFVFIIFGIV   175
tr|Q03NZ0|Q03NZ0_LACBA              LFKG----GQNVPSVILASVVSWGLTYIVNRGVESAAAMNTIITICKLIPLFTFIVVGIF   172
                                            . .: .::   :  * :  ::  *:: *: :*.:  . *::*:.      :

tr|A0A3M0SNP9|A0A3M0SNP9_9CLOT      MFKVNIFTLDFWGGSTP----------------SLGGVVAQVKSTMLVTLWVFIGIEGAV   217
tr|Q16C52|Q16C52_ROSDO              VFNWDTFSANFWGGADM----------------PEKSLLLQVRDTMLITVFVFIGIEGAS   173
sp|P18275|ARCD_PSEAE                AFKLDIFTADIWGKSNP----------------DLGSVMNQVRNMMLVTVWVFIGIEGAS   219
tr|G9A0G0|G9A0G0_RHIFH              AFETDLFRANFWGGAGM----------------PEASLFEQVRATMLVTVFVFIGVEGAS   218
tr|Q9CE19|Q9CE19_LACLA              LFKADVFTQHFWQTFTSNLAADGSVKSLVWHAMTAGGLLDQIKGSLMVMVWVFVGIEGAA   238
tr|B7VBS0|B7VBS0_LACFE              FFKAGMFTTDFWGHVANNLSKGT----------ETGSVYTQMKGTLLTLIWVFIGVEGAS   223
tr|G3ADI7|G3ADI7_LACSK              LFKGHLFTQAFWNNMSSSFVAG--------------DVMSQIKNCMMVMMWVFVGIEGAS   221
tr|Q03NZ0|Q03NZ0_LACBA              VFKGGMFTAHFWSNVSSGVAGGA-------------NIWTQFKSCLMIMMWVFVGVEGAS   219
                                     *:   *   :*                        .:  *.:  ::  ::**:*:***

tr|A0A3M0SNP9|A0A3M0SNP9_9CLOT      VVSGRAKRRSDVGKATVIGLVGTLVIYILITLLSLGIMNRARLSGLDTPSMAYVLESVVG   277
tr|Q16C52|Q16C52_ROSDO              VYSRYAKTRRDVGSATILGFVGVLGIMVAITLLPYGIMPQSAVADLRKPSLAGALEATVG   233
sp|P18275|ARCD_PSEAE                IFSSRAEKRSDVGKATVIGFITVLLLLVLVNVLSMGVMTQPELAKLQNPSMALVLEHVVG   279
tr|G9A0G0|G9A0G0_RHIFH              VYSRYARKRSDVGVATTLGFVGVLGLMVLVTLLPYAALERPEIAGMRQPSLASVLESVVG   278
tr|Q9CE19|Q9CE19_LACLA              MMGNRAKKKSDTAKATIIGLAVLLVIYVLLSLLPYGYMDQASLANVKAPGLVYILNEMVG   298
tr|B7VBS0|B7VBS0_LACFE              VMGHRAKNRTQAQQATIIGFILLSIIYVMISIIPYGTLNRAQLAAASQPALGNDLKLIVG   283
tr|G3ADI7|G3ADI7_LACSK              MLSARAEKKSDAGKATILGLVSLLAIYILASVLPYGYLTQDQLASIKQPAMLYIFEQMVG   281
tr|Q03NZ0|Q03NZ0_LACBA              MLSSRAKSKSEAGRATIIGIICLLIIYVLASVLPYGYLSQDALAKINQPAMLYIFQDMVG   279
                                    : .  *. : :.  ** :*:     : :  .::  . : :  ::    *.:   ::  **

tr|A0A3M0SNP9|A0A3M0SNP9_9CLOT      KWGAIVINLGLVISLLGATLGWTLLAAEIPYIAAKDGMFPKVFAKENKNGSAVNSLWITN   337
tr|Q16C52|Q16C52_ROSDO              ALETLFISIGVLISVLGAYLTWSLLVAEVPYAAAKSKDMPSVFGRENAQNVPANSLWLSN   293
sp|P18275|ARCD_PSEAE                HWGAVLISVGLLISLLGALLSWVLLCAEIMFAAAKDHTMPEFLRRENANQVPANALWLTN   339
tr|G9A0G0|G9A0G0_RHIFH              PWGSVFVSVGLIVSVLGAYLAWSLICVEVLFCAAKNGDMPSVLARENNNNVPAAALWLSN   338
tr|Q9CE19|Q9CE19_LACLA              GWGGSLMAIGLMISLLGAWLSWTMLPVEATQQLAEKKLLPSWFGKLNKYHAPSNSLLITQ   358
tr|B7VBS0|B7VBS0_LACFE              SWGATIINVGLIVSVIISWLSWTMLPAETTMLVAEDKAMPKVWGKLNAKKAPTASLMITG   343
tr|G3ADI7|G3ADI7_LACSK              TWGGYFIGVGLIISILGAWLSWTMLPAETMLLMAKQNLLPAYFGRVNKKKAPTFALVVTA   341
tr|Q03NZ0|Q03NZ0_LACBA              TWGGAFIGVGLIIAILGSWLSWTMLPADTTMLMAEEKLLPAYFGKKNKNGAPTFSLVLTA   339
                                         .: :*:::::: : * * :: .:     *:.  :*    : *       :* ::

tr|A0A3M0SNP9|A0A3M0SNP9_9CLOT      ILVEISLILTLFSSS------TYQILYSIASGAILIPYFLSALFGLKF----ELMSKEEN   387
tr|Q16C52|Q16C52_ROSDO              SIISVFLISTYWSAD------AFNFMLDMATVASLLPYSLVAGYGILLTRSNEIYGDEQG   347
sp|P18275|ARCD_PSEAE                ICVQVFLVVVFFTSGDPDGMDPYTKMLLLATSMILIPYFWSAAYGLLLTLKGETYENDAR   399
tr|G9A0G0|G9A0G0_RHIFH              GVIQFFLVSTLFSEN------AFRLMVNLTSAMVLVPYLLVAAYGFLIARRGETYHIRPE   392
tr|Q9CE19|Q9CE19_LACLA              LMIQIFIIITYFVAN------AYNVFIYMATAVIMICYALVGAYLLKIGI----KE----   404
tr|B7VBS0|B7VBS0_LACFE              VLQTIFLFSLLFTEQ------AYNFAYSLASAAILFSYLLVGLYQMKYSQ----EHQ---   390
tr|G3ADI7|G3ADI7_LACSK              GLIQVFLFTLLFTTK------AYNFAYSLCTASIIVCYMLVAAYQIKYSW----AHLQEK   391
tr|Q03NZ0|Q03NZ0_LACBA              ALIQIFLLVLLFSEE------AYNFALSLCTAAIVVCYIFVGAYQVKFSY----QNK---   386
                                        . :.   :          :     : :   :. *   . : .

tr|A0A3M0SNP9|A0A3M0SNP9_9CLOT      GRTKNIIIASVATIYTAWLVYAAGLKYVLLETILFAIGIVAFTIASKENNSNKKKNYIFL   447
tr|Q16C52|Q16C52_ROSDO              KQKRDSIFAWVAAVYVVFMFVAAGLKYIILVAVIYAPLTVLYFWARKEQNLP-----YFT   402
sp|P18275|ARCD_PSEAE                ERSKDLVIAGIAVAYAVWLLYAGGLKYLLLSALLYAPGAILFAKAKHEVGQP-----IFT   454
tr|G9A0G0|G9A0G0_RHIFH              ERFSDLIFAGAATVYTAFMIYAGGLELLLLSTILYAFGTVLFLYTRREQKKP-----LFN   447
tr|Q9CE19|Q9CE19_LACLA              GSIKNILIGFFTFAFQALALYLSGWQYVWLAMILYTIGFILFIKAKKESRQAIS---VKE   461
tr|B7VBS0|B7VBS0_LACFE              -EWGQWTIGLLLVLFQIACMFLAGWQQVLLVSISFIPGFIIYYQGVRENNRTMS---GGE   446
tr|G3ADI7|G3ADI7_LACSK              GNRQQLLIGVLALLFEIAGILMAGVSYLLLCFIAYIPGIYFYGRARKNNGHQH----FLS   447
```

```
tr|Q03NZ0|Q03NZ0_LACBA              -DMKQFWIGFFALLFQVVAITLAGLHFLMLVCIGYLPGIYFYYRAKKDYSLDGG---KLT   442
                                        :  :.     :    .  .*   : *  : :      :    ::

tr|A0A3M0SNP9|A0A3M0SNP9_9CLOT      SYEKVIALIFLVAGIVAVVMLATGKLSIS----------   476
tr|Q16C52|Q16C52_ROSDO              KVELIVFGIVLLVAAIGVYGLFSGAITP-----------   430
sp|P18275|ARCD_PSEAE                GIEKLIFAAVVIGALVAAYGLYDGFLTL-----------   482
tr|G9A0G0|G9A0G0_RHIFH              PREWLVFMAAAVGCVFGIYSLVTGYITL-----------   475
tr|Q9CE19|Q9CE19_LACLA              WAGM---FIVALLGVLAILVLICGAKSGTALDLRGLLGY   497
tr|B7VBS0|B7VBS0_LACFE              KGTM---VVVLALSLIAIFLIFNGTIAVG----------   472
tr|G3ADI7|G3ADI7_LACSK              KGEWLITTIIVIGAIIGIWLVVSGKIVI-----------   475
tr|Q03NZ0|Q03NZ0_LACBA              KMEILYSIAIVAFAIISIVMVAMGAIQI-----------   470
                                                   ..   :  *
```

ArcD homologues, matrix with percentage identity

1:	tr|A0A3M0SNP9|A0A3M0SNP9_9CLOT	100.00	38.26	51.28	42.13	38.44	38.58	39.35	36.80
2:	tr|Q16C52|Q16C52_ROSDO	38.26	100.00	44.42	50.00	31.10	29.36	32.23	31.58
3:	sp|P18275|ARCD_PSEAE	51.28	44.42	100.00	52.00	36.21	33.91	40.38	38.10
4:	tr|G9A0G0|G9A0G0_RHIFH	42.13	50.00	52.00	100.00	30.45	30.67	35.12	31.39
5:	tr|Q9CE19|Q9CE19_LACLA	38.44	31.10	36.21	30.45	100.00	39.79	45.18	44.64
6:	tr|B7VBS0|B7VBS0_LACFE	38.58	29.36	33.91	30.67	39.79	100.00	49.25	48.18
7:	tr|G3ADI7|G3ADI7_LACSK	39.35	32.23	40.38	35.12	**45.18**	49.25	100.00	64.10
8:	tr|Q03NZ0|Q03NZ0_LACBA	36.80	31.58	38.10	31.39	44.64	48.18	64.10	100.00

ArcE homologues, amino acid sequence alignment

```
tr|Q9CE19|Q9CE19_LACLA              ------------------------------------------------------------   0
tr|A0A0H2ZNX2|A0A0H2ZNX2_STRP2      ------MSEKAKKGFKMPSSYTVLLIIIAIMAVLTWFIPAGAFIEGIYETQPQN-----P   49
tr|G3ADI9|G3ADI9_LACSK              MDDAEIIKTKKRFKLKMPGAFVILFILTVVAVMATWVVPSGSYAKLSYDQKSTQLVVTKP   60
tr|Q03NY7|Q03NY7_LACBA              MANAEVQPPKKKFKLKMPGAFVILFILTILAVMATWVIPAGSYAKLTYNQATSSLQIKSP   60


tr|Q9CE19|Q9CE19_LACLA              ------------------------------------------------------------   0
tr|A0A0H2ZNX2|A0A0H2ZNX2_STRP2      QGIWDVLMA----------PI------------------------RAMLGTHPEEGSLIK   75
tr|G3ADI9|G3ADI9_LACSK              SGQVEKVPATQASLDRLGVKIKISQFTSGSINAAVSIPNTYQRLKQRPASLAAVPNSMVR   120
tr|Q03NY7|Q03NY7_LACBA              TGKTTRAPATQKELDRLGVKINIDQFTSGGISEAVSIPNTYQRLKQRPASLWAVTGSMVR   120


tr|Q9CE19|Q9CE19_LACLA              ---------------------------------MENKKTKGISLFALLAIIISGAIGGGV   27
tr|A0A0H2ZNX2|A0A0H2ZNX2_STRP2      ETSAAIDVAFFILMVGGFLGIVNKTGALDVGIASIVKKYKGREKMLILVLMPLFALGGTT   135
tr|G3ADI9|G3ADI9_LACSK              GTVEAVDIMVFILVLGGLIGTVKASGAFESGLLALTKKTKGHEFLLIFFVAILMVLGGTL   180
tr|Q03NY7|Q03NY7_LACBA              GTIEAVDIMVFIFVLGGLIGVVKASGAFESGLMALTKKTKGHEFLLIFLVAILMVLGGTL   180
                                                                    ** ** . : :: :    .:**

tr|Q9CE19|Q9CE19_LACLA              FNLANDLANGSTPGGVMISWLFIGF------GIFMLVLSFNRL-ITIRPKLSGVSDYARE   80
tr|A0A0H2ZNX2|A0A0H2ZNX2_STRP2      YGMGEETMAF-YPLL-VPVMMAVGFDSLTGVAIILLGSQIGCLASTLNPFATGI-ASATA   192
tr|G3ADI9|G3ADI9_LACSK              CGIEEEAVAF-YPIL-VPVFIAMGYDSIVCVGAIFLASSIGTTFSTINPFSVVI-ASNAA   237
tr|Q03NY7|Q03NY7_LACBA              CGIEEEAVAF-YPIL-VPIFIAMGYDSIVCVGAIFLASSVGTAFSTINPFSAVI-ASNAA   237
                                     .: ::      *   :   : :*:      . ::*  ...    *:.*    :

tr|Q9CE19|Q9CE19_LACLA              GFGDFIGFLSGWGYWISAWTGTIGFAVLMMTSADYFFPSKFANSNGSLTVLSVIIVSIIS   140
tr|A0A0H2ZNX2|A0A0H2ZNX2_STRP2      GVGTGDGIVLRLIFWVTLT----ALS----TWFVYRYADKIQKDPTK----S--------   232
tr|G3ADI9|G3ADI9_LACSK              GISFTEGLLWRVGGCIVGA----IFV----IYYLYRYSKKVKADPTQ----S--------   277
tr|Q03NY7|Q03NY7_LACBA              GIDFTQGIVWRIIGLIVAA----IFL----IFYLYWYSKKVKNQPTF----S--------   277
                                    *..   *::      :        :         *  :  .*.  .       *

tr|Q9CE19|Q9CE19_LACLA              WILMFLVDRGVETAALVNAVVMIAKLIPLVVFSITGIILFKADVFTQHFWQTFTSNLAAD   200
tr|A0A0H2ZNX2|A0A0H2ZNX2_STRP2      -------------------------------------LVYSTRKEDLKHFNVEES-SSV-   253
tr|G3ADI9|G3ADI9_LACSK              -------------------------------------YSYEDHDAFDKMWAITSEGAQS-   299
tr|Q03NY7|Q03NY7_LACBA              -------------------------------------YTYEDRESFNHMWSVASTNDQI-   299
                                                                           :.      : :

tr|Q9CE19|Q9CE19_LACLA              GSVKSLVWHAMTAGGLLDQIKGSLMVMVWVFVGIEGAAMMGNRAKKKSDTAKATIIGLAV   260
tr|A0A0H2ZNX2|A0A0H2ZNX2_STRP2      --ESTLSSKQ---KSVLFLFVLTFILMVLSF-----------------------------   279
tr|G3ADI9|G3ADI9_LACSK              --KAVFTWRK---KLILVLFVVTFPIMVWGV-----------------------------   325
tr|Q03NY7|Q03NY7_LACBA              --KTGFSMRK---KLILTLFVVTFPIMVWGV-----------------------------   325
                                         :  :      :*  :  :: :**  .

tr|Q9CE19|Q9CE19_LACLA              LLVIYVLLSLLPYGYMDQASLANVK--APGLVY------ILNEMVGGWGGSLMAI-----   307
tr|A0A0H2ZNX2|A0A0H2ZNX2_STRP2      ----------IPWTDLGVTIFDDFNAWLTGLPVIGNIVGSSTSALGTWYFPEGAMLFAFM   329
tr|G3ADI9|G3ADI9_LACSK              ----------M---------------------------------SQGWWFPTMAASFLTF   342
tr|Q03NY7|Q03NY7_LACBA              ----------M---------------------------------SQGWWFPTMASSFLVF   342
                                              :                                    *     *

tr|Q9CE19|Q9CE19_LACLA              GLMISLLGAWLSWTMLPVEATQQLAEKKLLPSWFGKLNKYH-------------------   348
tr|A0A0H2ZNX2|A0A0H2ZNX2_STRP2      GILIG--------------VIYGLKEDKIISSFMNGAADLLSVALIVAIARGIQVIMNDG   375
tr|G3ADI9|G3ADI9_LACSK              AIIIM---------FLTATGPEGIGEKGVIDAFVNGASSLVGVSLIIGLARGINLIMNEG   393
tr|Q03NY7|Q03NY7_LACBA              AIIIM---------FLTATGPNGLGEKGVVDAFVAGASSLVGVSLIIGLARGINLVMNEG   393
                                    .::*                   : *. :: ::.    .
```

ArcE homologues, matrix with percentage identity

1: tr|Q9CE19|Q9CE19_LACLA	100.00	18.32	19.87	21.73
2: tr|A0A0H2ZNX2|A0A0H2ZNX2_STRP2	18.32	100.00	33.76	34.75
3: tr|G3ADI9|G3ADI9_LACSK	19.87	33.76	100.00	79.38
4: tr|Q03NY7|Q03NY7_LACBA	21.73	34.75	79.38	100.00

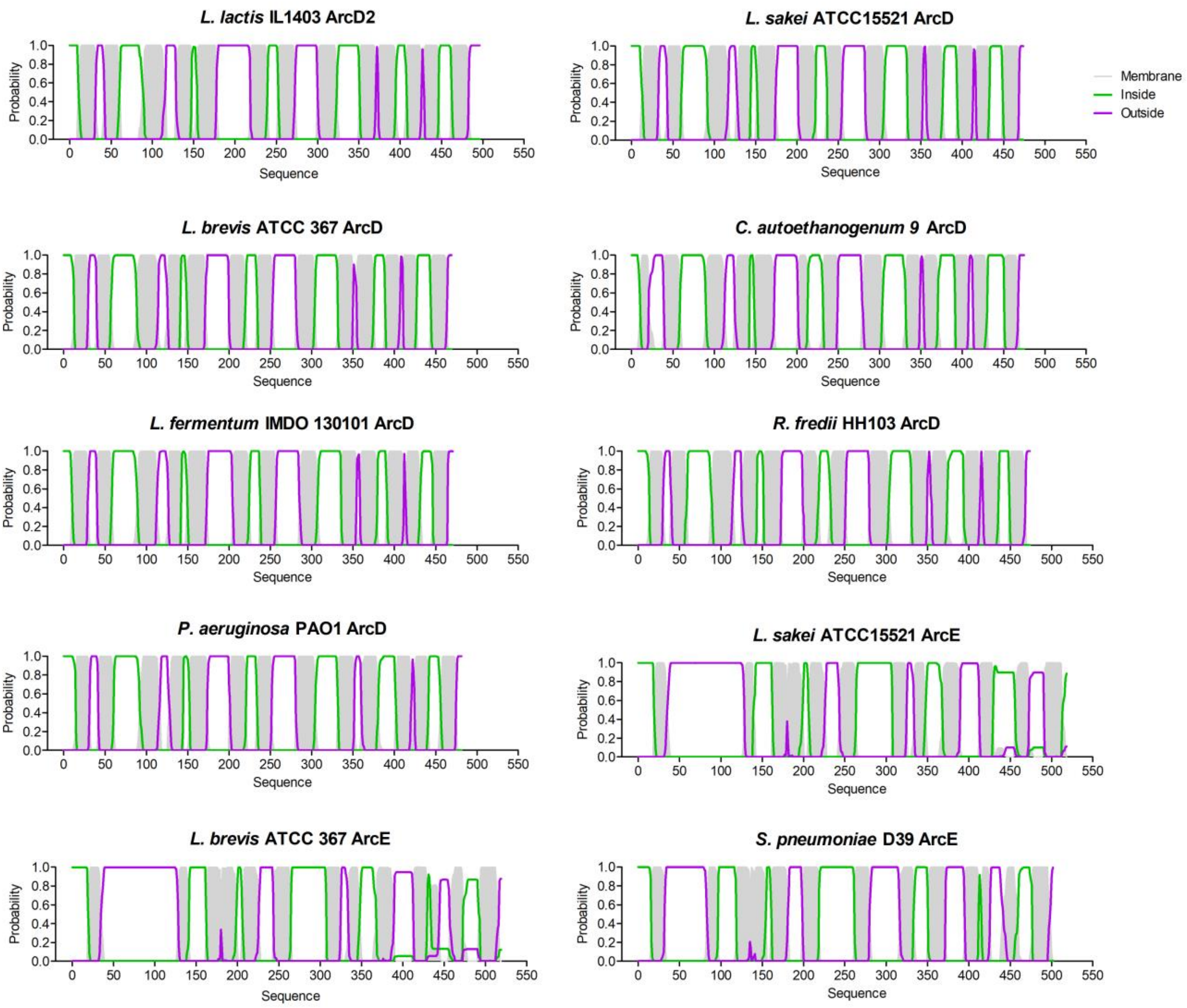

Figure S2. Predicted topology of ArcD and ArcE proteins. The predicted topology[3] of ArcD and ArcE proteins is shown for internal (green) and external (purple) loop regions and transmembrane (grey) segments.

Table S2. Plasmids used in this study

Plasmid	Host	Description	Reference
FX cloning plasmids	*E. coli, L. lactis*	(Addgene Kit # 1000000039)	4
pINIT_cat	*E. coli*	Sequencing vector, Cm^R	4
pERL	*E. coli*	Exchange vector with pREX constructs, Ery^R	5
pREx-His(NH3)	*E. coli*	Expression vector (VBEx), Amp^R, P_{NisA}, 10x N-terminus His-tag	4
pREx-His(C3H)	*E. coli*	Expression vector (VBEx), Amp^R, P_{NisA}, 10x C-terminus His-tag	4
pNZ*arcD*	*L. lactis*	Template vector encoding *arcD* from *L. brevis* ATCC 367	6
pNZ*arcEpn*	*L. lactis*	Template vector encoding *arcE* from *S. pneumoniae* D39	7
pNZ*arcD-CA*	*L. lactis*	Expression vector, Cm^R, *arcD* from *C. autoethanogenum* 9, P_{NisA}, 10x C-terminus His-tag	This study
pNZ*arcD-LB*	*L. lactis*	Expression vector, Cm^R, *arcD* from *L. brevis* ATCC 367, P_{NisA}, 10x C-terminus His-tag	This study
pNZ*arcD-LF*	*L. lactis*	Expression vector, Cm^R, *arcD* from *L. fermentum* IMDO 130101, P_{NisA}, 10x C-terminus His-tag	This study
pNZ*arcD-LS*	*L. lactis*	Expression vector, Cm^R, *arcD* from *L. sakei* ATCC15521, P_{NisA}, 10x N-terminus His-tag	This study
pNZ*arcD-PA*	*L. lactis*	Expression vector, Cm^R, *arcD* from *P. aeruginosa* PAO1, P_{NisA}, 10x C-terminus His-tag	This study
pNZ*arcD-RF*	*L. lactis*	Expression vector, Cm^R, *arcD* from *R. fredii* HH103, P_{NisA}, 10x C-terminus His-tag	This study
pNZ*arcD-RD*	*L. lactis*	Expression vector, Cm^R, *arcD* from *R. denitrificans* ATCC 33942, P_{NisA}, 10x C-terminus His-tag	This study
pNZ*arcE-LB*	*L. lactis*	Expression vector, Cm^R, *arcE* from *L. brevis* ATCC 367, P_{NisA}, 10x C-terminus His-tag	This study
pNZ*arcE-LS*	*L. lactis*	Expression vector, Cm^R, *arcE* from *L. sakei* ATCC15521, P_{NisA}, 10x C-terminus His-tag	This study
pNZ*arcE-SP*	*L. lactis*	Expression vector, Cm^R, *arcE* from *S. pneumoniae* D39, P_{NisA}, 10x N-terminus His-tag	This study
pNZ*arcD2ΔC*	*L. lactis*	Expression vector, Cm^R, *arcD2* from *L. lactis* IL1403, P_{NisA}, 10x C-terminus His-tag	8
pNZ*arcA*	*L. lactis*	Expression vector, Cm^R, *arcA* from *L. lactis* IL1403, P_{NisA}, 10x C-terminus His-tag	8
pNZ*arcB*	*L. lactis*	Expression vector, Cm^R, *arcB* from *L. lactis* IL1403, P_{NisA}, 10x C-terminus His-tag	8
pNZ*arcC1*	*L. lactis*	Expression vector, Cm^R, *arcC1* from *L. lactis* IL1403, P_{NisA}, 10x N-terminus His-tag	8
pRsetA*glpK*	*E. coli*	Expression vector, Amp^R, *glpK* from *E. coli* BL21-DE3, P_{T7}, 6x N-terminus His-tag	9
pBADmycHisB-*glpT*	*E. coli*	Expression vector, Amp^R, *glpT* from *E. coli* DH5α, P_{BAD}, 6x N-terminus His-tag	10
pRSF-Duet-1*fadD* (pME001)	*E. coli*	Expression vector, Kan^R, *fadD* from *E. coli* MG1655, P_{T7}, 6x C-terminus His-tag	11
pET-28b*plsB* (pME002)	*E. coli*	Expression vector, Kan^R, *plsB* from *E. coli* MG1655, P_{T7}, 6x C-terminus His-tag	11
pET-28b*plsC* (pME003)	*E. coli*	Expression vector, Kan^R, *plsC* from *E. coli* MG1655, P_{T7}, 6x C-terminus His-tag	11
pBADPercevalHR	*E. coli*	Expression vector, Amp^R, PercevalHR, P_{BAD}, 7x N-terminus His-tag	9

Table S3. Primers used in this study

Organism	Name of primer	Sequence of primer (5’ to 3’)
Pseudomonas aeruginosa PAO1	arcD Fw	ATATATGCTCTTCTAGTTCCCAAGAATCCAGCCAAAAAC
	arcD Rv	TATATAGCTCTTCATGCGAGGGTGAGGAAACCGTCGTAG
Lactobacillus sakei ATCC15521	arcD Fw	ATATATGCTCTTCTAGTACGGAGGAAAAACCAGCAAAAAAAATTG
	arcD Rv	TATATAGCTCTTCATGCAATAACGATTTTCCCTGAAACAAC
Lactobacillus sakei ATCC15521	arcE Fw	ATATATGCTCTTCTAGTGATGATGCTGAAATAATCAAGAC
	arcE Rv	TATATAGCTCTTCATGCCGAATAAATTAGGACTTGTAAG
Roseobacter denitrificans ATCC 33942	arcD Fw	TATATAGCTCTTCATGCAATAACGATTTTCCCTGAAACAAC
	arcD Rv	TATATAGCTCTTCATGCGGAGTGATAGCACCCGAAAAAAG
Clostridium autoethanogenum 9	arcD Fw	ATATATGCTCTTCTAGTTCAGAAAATAATAAATTGGGG
	arcD Rv	TATATAGCTCTTCATGCACTTATTGACAATTTACCTGTTG
Streptococcus pneumoniae D39	arcE Fw	ATATATGCTCTTCTAGTAGTGAAAAAGCTAAAAAAGGG
	arcE Rv	TATATAGCTCTTCATGCTAGAAAAGGGAGGAAGGTTCCAAGGAG
Rhizobium fredii HH103	arcD Fw	ATATATGCTCTTCTAGTAGTGAGGAAACAAAAAAAGGG
	arcD Rv	TATATAGCTCTTCGTGCACCAATAGCTGGAATGAAGGTAC
Streptococcus gordonii ATCC 35105	arcD Fw	ATATATGCTCTTCTAGTGCTAATGCTGAAGTTCAACCACC
	arcD Rv	TATATAGCTCTTCCTGCAACACCTGAAATTAATACTTC
Lactobacillus brevis ATCC 367	arcE Fw	ATATATGCTCTTCTAGTCTAAATCTGGATAGCACCCATCGCC
	arcE Rv	TATATAGCTCTTCGTGCATGGATGAAAATAAAGGCTTATCAATG
Lactobacillus fermentum IMDO 130101	arcD Fw	ATATATGCTCTTCTAGTACATCAACTGCTCAAAAACTTAGTC
	arcD Rv	TATATAGCTCTTCATGCTAAAGTAATATAACCAGTAACTAATG

Figure S3. Nucleotide sequence of *arcD and arcE* genes. Nucleotide sequence of expression vectors used in this study. Color code: P_{NisA} promoter region (grey); translation initiation codon (green); *arcD* and *arcE* sequences (orange); HRV C3 cleavage site (blue); 10x-His tag (purple); stop codon (red).

pNZ*arcD-CA* (*arcD Clostridium autoethanogenum* 9)

5’CTAGTCTTATAACTATACTGACAATAGAAACATTAACAAATCTAAAACAGTCTTAATTCTATCTTGAGAAAGTATTGGTAATAATATTA
TTGTCGATAACGCGAGCATAATAAACGGCTCTGATTAAATTCTGAAGTTTGTTAGATACAATGATTTCGTTCGAAGGAACTACAAAAT
AAATTATAAGGAGGCACTCACCATGAGTTCAGAAAATAATAAATTGGGGTTATTTTCACTTATAGCATTAGTTATCGGCTCAATGATT
GGTGGAGGAGCATTTTCGCTGCCAGGTGATATGGCAAAAGGAGCTAGTGCAGGTGCAATTATTATAGGATGGTTAATTACTGGAAT
TGGTATGATAGCATTAGCTTTTGTATATCAAAATCTTTCAATGAAAAGACCAGATTTAAATGGTGGTATATATAGTTATGCTAAAGCTG
GTTTTGGAGGTTACATGGGATTTAATTCAGCTTGGGGATATTGGTTAAGTGCATTAATTGGTAATGTATCTTATTTGGTAATGATGTTT
GGAGCTGTTGGATACTTTTTTCCTGTATTCGGAAAAGGAAATAATTTAGCATCTGTTGTTTGCGCATCTATTATGTTGTGGCTTATTCA
AGGTTTGATATTAAAAGGAGTAAAACAAGCTGCTATAGTAAATGTTATCACTACAATTGCAAAGTTAGTGCCAATATTCCTATTTGTAA
TTATAGCTATCATAATGTTTAAAGTTAATATATTTACCTTAGATTTTTGGGGTGGTTCTACTCCTAGCCTAGGGGGAGTTGTAGCTCAA
GTTAAAAGTACCATGTTAGTAACATTATGGGTTTTTATAGGAATTGAAGGAGCTGTAGTTGTTTCAGGTAGAGCTAAAAGAAGAAGTG
ATGTAGGAAAAGCTACAGTAATAGGCTTGGTTGGAACATTAGTCATATATATTTTAATAACACTATTATCTTTAGGAATAATGAATAGG
GCTCGCCTTTCTGGTTTAGACACTCCATCAATGGCATATGTACTTGAAAGTGTTGTTGGAAAGTGGGGAGCAATCGTAATAAATTTA
GGATTGGTAATTTCTTTGTTAGGAGCAACTTTAGGTTGGACTCTTTTGGCAGCAGAAATTCCGTATATAGCTGCAAAAGATGGTATGT
TCCCGAAAGTATTTGCTAAAGAAAATAAAAATGGATCAGCAGTAAATTCTTTATGGATTACTAATATATTAGTAGAAATTTCACTTATA
CTAACACTGTTTTCTAGTAGTACCTATCAAATACTATATTCAATAGCAAGTGGAGCTATATTAATTCCTTATTTTCTTAGTGCACTGTTT
GGGCTAAAATTTGAATTAATGAGTAAAGAAGAAAATGGAAGAACTAAAAATATTATAATTGCATCAGTTGCAACTATATACACAGCAT
GGTTAGTATATGCAGCAGGGTTAAAATACGTTTTGTTAGAAACTATACTTTTTGCTATTGGCATAGTTGCATTTACAATTGCCAGCAAA
GAGAACAATAGTAATAAGAAGAAAAATTATATTTTCTTATCATATGAGAAAGTGATAGCACTAATTTTCTTAGTAGCAGGAATAGTTGC
AGTAGTTATGCTAGCAACAGGTAAATTGTCAATAAGTGCATTAGAAGTTTTGTTTCAAGGTCCACACCATCATCATCACCATCATCAT
CATCATTAA3’

pNZ*arcD-LB* (*arcD Lactobacillus brevis* ATCC 367)

5’CTAGTCTTATAACTATACTGACAATAGAAACATTAACAAATCTAAAACAGTCTTAATTCTATCTTGAGAAAGTATTGGTAATAATATTA
TTGTCGATAACGCGAGCATAATAAACGGCTCTGATTAAATTCTGAAGTTTGTTAGATACAATGATTTCGTTCGAAGGAACTACAAAAT
AAATTATAAGGAGGCACTCACCATGAGTCTAAATCTGGATAGCACCCATCGCCACCATTACAATACTAATAATGGCAAAGGCCACAA
TGGCAATCGAATACAAAATTTCCATTTTAGTCAACTTACCACCATCGAGACTATAATCTTTCTTAGCGCGATAATAGAAGTAAATCCCT
GGTAAGTAACCGATACATACCAACATTAAGAAGTGTAAACCGGCTAAGGTAATCGCAACAACCTGGAACAAGAGTGCGAAGAAGCC
AATCCAAAATTGCTTCATGTCTTTGTTCTGATAAGAGAACTTCACTTGATAGGCTCCAACGAAGATGTAGCAAACCACAATAGCAGCC
GTACACAAGGATAATGCAAAGTTATAAGCTTCTTCGGAGAATAGAAGAACTAACAGGAAAATTTGGATCAAAGCCGCCGTCAAGACT
AGAGAAAAGGTTGGCGCACCATTTTTATTCTTCTTGCCAAAGTAAGCTGGTAAGAGTTTTTCTTCTGCCATTAACATCGTTGTATCAG
CAGGGAGCATCGTCCAAGATAACCATGACCCAAGGATAGCAATGATCAGGCCGACACCAATGAAGGCCCCACCCCAAGTACCAAC
CATGTCTTGGAAAATATATAACATTGCTGGCTGGTTGATCTTAGCCAAAGCGTCTTGACTTAAGTAACCGTATGGCAAAACTGAAGCT
AATACATAAATAATCAGTAGGCAAATGATCCCAATGATCGTGGCCCGACCAGCTTCACTCTTACTCTTGGCCCGAGAAGATAGCATG
CTGGCTCCTTCAACCCCAACGAAGACCCACATCATAATCATTAAACAAGACTTGAATTGTGTCCAAATATTGGCGCCACCTGCTACA
CCGGAAGATACATTTGACCAGAAGTGAGCGGTGAACATACCACCTTTGAAGACAAAGATACCAACAACAATGAAGGTAAACAGTGG
AATTAACTTACAAATTGTGATAATCGTATTCATCGCTGCCGCAGATTCAACCCCGCGGTTAACGATATAGGTTAGTCCCCACGAAAC
CACACTGGCCAAGATAACTGATGGGACATTTTGACCACCTTTGAACAGCGGGAAGAAATAACCCAGTGAACTCATCAGAACTGTCG
CAAAGGCCACGTTACCTAACCAAGCGCTAAGCCAATATCCCCAGCCACTGACAAATCCAGCAAAATTACCAAATCCGGCTTGTGCAT
ACGCCTGAACACCTTCAGCTTCCGGATTCTTTTGAAGTAAATTATTTAAAGATAACGCTAACATTAAGATCCCGAAACCAACGACTAA
CCAGGCCAGCAGCACTGGCCCAGCAGCTGCCCCGCCGGCTAATGAACTGGTCAGTGTGAAGACCCCAGAACCAATTGAACTGGTA
ACAACGGCGGCAGTTAGCGCACCCATTGATAAGCCTTTATTTTCATCCATTGCATTAGAAGTTTTGTTTCAAGGTCCACACCATCATC
ATCACCATCATCATCATCATTAA3’

pNZ*arcD-LF* (*arcD Lactobacillus fermentum* IMDO 130101)

5’CTAGTCTTATAACTATACTGACAATAGAAACATTAACAAATCTAAAACAGTCTTAATTCTATCTTGAGAAAGTATTGGTAATAATATTA
TTGTCGATAACGCGAGCATAATAAACGGCTCTGATTAAATTCTGAAGTTTGTTAGATACAATGATTTCGTTCGAAGGAACTACAAAAT
AAATTATAAGGAGGCACTCACCATGAGTGAAAACAAAAACGGAATTGGGCGCATTGGCCTAATTGCCCTGATCGTCAGTTCCTGTAT
CGGGACGGGGATCTTTGGGATCACCAATGCGGTGGCCGCCGCGGCGGCACCTGGGCCCGCCCTACTAGCATGGCTCTTCGTCGG
CTTTGGCTTTTTGATGCTCGTCTTTGCGCTTAACAACCTATCGGAAAAACGGCCGGACCTGGAAGCGGGTATCTTCTCATATGCCGG
TGCCGGCTTTGGCCGATGGGGGAATTTATTTCCGGTTGGGCCTACTGGTTGTCAGCTTGGTTGGGTAACATTGCCTTTGCCACCA
TGCTGATGAGTGCCTTGGGAACCTTTTTCCCGGTCTTTGGCAACGGGCAGAACCTAACGTCGATCATCGTGGCCATTATCTTCTGTT
GGGTTTTGACCCTGTTGGTTAACAGCGGGGTGGAAACGGCGACCTTTATTAACACGATTGGGACCTTCTTTAAGATCATTCCACTGG
TATTATTCATCATTATTTCGATCATCTTCTTTAAGGCCGGTATGTTTACGACCGACTTCTGGGGCCACGTCGCTAACAACCTTTCCAA
GGGGACGGAAACGGGCTCGGTATACACCCAGATGAAGGGGACCCTACTAACACTGATCTGGGTCTTCATCGGGGTTGAAGGGGC
CTCCGTTATGGGTCACCGGGCCAAGAACCGGACCCAAGCCCAACAGGCGACGATCATCGGGTTCATCCTCTTGTCGATCATTTACG

TGATGATTTCGATTATTCCTTACGGGACTCTGAACCGGGCCCAACTGGCCGCCGCTAGTCAACCGGCCCTGGGGAACGACTTGAA
GTTGATCGTTGGTTCCTGGGGGGCCACGATTATCAACGTCGGCTTAATTGTTTCGGTAATTATTTCCTGGCTTTCCTGGACGATGCT
GCCAGCCGAAACGACGATGCTAGTTGCCGAAGACAAGGCAATGCCAAAGGTTTGGGGTAAACTCAACGCTAAGAAGGCCCCAACG
GCCTCGCTGATGATCACTGGGGTTTTGCAAACGATCTTCCTGTTCTCCTTACTCTTCACTGAACAAGCCTACAATTTTGCTTACTCAC
TGGCCTCCGCCGCCATCCTCTTCTCCTATCTTTTGGTGGGGCTATACCAAATGAAGTACAGTCAAGAACACCAAGAGTGGGGACAG
TGGACGATTGGACTCTTGTTAGTTCTCTTCCAAATTGCCTGCATGTTCTTGGCCGGTTGGCAACAAGTGCTCCTGGTTTCGATTAGC
TTTATTCCGGGCTTTATCATCTACTACCAAGGGGTACGTGAAAACAACCGGACGATGAGCGGGGGCGAAAAGGGAACGATGGTCG
TAGTCTTAGCCCTTAGTTTAATTGCCATCTTCCTGATCTTTAACGGAACGATTGCTGTCGGGGCATTAGAAGTTTTGTTTCAAGGTCC
ACACCATCATCATCACCATCATCATCATCATTAA3’

pNZ*arcD-LS* (*arcD Lactobacillus sakei* ATCC15521)

5’CTAGTCTTATAACTATACTGACAATAGAAACATTAACAAATCTAAAACAGTCTTAATTCTATCTTGAGAAAGTATTGGTAATAATATTA
TTGTCGATAACGCGAGCATAATAAACGGCTCTGATTAAATTCTGAAGTTTGTTAGATACAATGATTTCGTTCGAAGGAACTACAAAAT
AAATTATAAGGAGGCACTCACCATGCACCATCATCATCACCATCATCATCATCATTTAGAAGTTTTGTTTCAAGGTCCAAGTATGACG
GAAGAAAAACCAGCAAAAAAAATTGGCTTATTGGCATTAATTGCATTGGTTATTAGTTCATCTATTGGTAGTGGGGTCTTCGGATTAA
CATCTGATTTAGCGAGTGCATCCGCACCGGGCCCAGTGTTAATCGCATGGGTGATTGTCGGCTTTGGGATTTTAATGCTGGCATTAT
CCTTGAATAATTTATTGATGAAAGAACCTGAACTAGAAGGGATTTTCTCTTACGCTGAAAAGGGCTTTGGCCCCTTTGCCGGCTTCAT
TAGTGGCTGGGGTTATTGGTTGTCAGCATGGTTGGGGAACGTAGCTTTTGCGACAATTTTAATGAGTGCGTTGGGTTATTTCTTCCC
GATTTTTAAATCAGGGCAGAACTTACCCTCAATCTTAGTTGCCAGTGTTTTATCGTGGAGCTTAACATATTTTGTTAATCGCGGGGTA
GAAGGCGCAGCTGCGATCAATACATTGGTAACAATTTGTAAATTGATTCCACTATTTGTCTTCATTATTTTTGGGATTGTGTTATTTAA
AGGTCATCTTTTCACACAAGCATTTTGGAATAATATGAGTAGTAGCTTTGTTGCCGGCGATGTGATGAGCCAAATCAAGAACTGTAT
GATGGTTATGATGTGGGTCTTTGTCGGTATCGAAGGCGCTTCAATGTTATCGGCGCGGGCGGAAAAGAAGTCTGATGCTGGTAAAG
CAACAATTTTAGGATTAGTGAGTTTACTAGCCATTTATATCTTAGCGTCTGTTTTACCTTATGGGTATTTAACGCAAGATCAATTAGCT
AGCATCAAGCAACCAGCGATGTTATACATTTTTGAACAGATGGTTGGGACTTGGGGGGGCTACTTCATCGGTGTTGGGTTAATCATC
TCAATTTTAGGGGCTTGGTTATCTTGGACAATGTTACCTGCCGAAACAATGTTGTTAATGGCAAAGCAAAATTTATTGCCAGCTTACT
TCGGTCGCGTCAACAAGAAAAAAGCACCGACGTTTGCACTAGTCGTGACAGCGGGGTTGATTCAAGTTTTCCTATTCACCCTATTAT
TCACAACGAAAGCTTATAACTTCGCTTATTCATTATGTACGGCTTCAATTATCGTTTGTTACATGTTGGTGGCAGCTTATCAAATTAAA
TATTCTTGGGCCCACCTGCAAGAAAAAGGAAATCGGCAACAATTATTAATTGGGGTTTTAGCCTTATTATTTGAAATTGCCGGAATCT
TAATGGCGGGTGTCAGTTACTTATTACTCTGTTTTATAGCCTATATTCCGGGGATTTACTTCTATGGTCGTGCCCGTAAAAATAATGG
GCACCAACATTTCTTATCAAAAGGGGAATGGTTAATCACGACAATTATTGTCATCGGTGCAATTATCGGGATTTGGTTAGTTGTTTCA
GGGAAAATCGTTATTTAG3’

pNZ*arcD-PA* (*arcD Pseudomonas aeruginosa* PAO1)

5’CTAGTCTTATAACTATACTGACAATAGAAACATTAACAAATCTAAAACAGTCTTAATTCTATCTTGAGAAAGTATTGGTAATAATATTA
TTGTCGATAACGCGAGCATAATAAACGGCTCTGATTAAATTCTGAAGTTTGTTAGATACAATGATTTCGTTCGAAGGAACTACAAAAT
AAATTATAAGGAGGCACTCACCATGAGTTCCCAAGAATCCAGCCAAAAACTCCGACTAGGAGCGCTAACGGCTCTGGTTGTCGGCT
CGATGATCGGAGGGGGCATCTTCTCCCTCCCGCAAAACATGGCAGCCAGCGCCGATGTCGGCGCCGTCCTGATCGGATGGGCGA
TCACCGCCGTCGGCATGCTCACCCTCGCTTTCGTCTTCCAGACCCTCGCCAACCGCAAGCCCGAACTCGACGGCGGCGTGTACGC
CTACGCCAAGGCCGGCTTCGGCGACTACATGGGTTTCTCCTCGGCCTGGGGCTACTGGATCAGCGCCTGGCTGGGCAACGTCGG
CTACTTCGTCCTGTTGTTCAGCACCCTCGGCTACTTCTTCCCGATCTTCGGCAAGGGCGACACCGTCGCGGCGATCGTCTGCGCCT
CGGTCCTGCTCTGGGCCCTGCACTTCCTGGTGCTGCGCGGGATCAAGGAGGCGGCGTTCATCAACACCGTCACCACCGTGGCCA
AGGTCGTGCCGCTGTTCCTGTTCATCCTGATCTGCCTGTTCGCCTTCAAGCTGGACATCTTCACCGCCGACATCTGGGGCAAGAGC
AACCCGGACCTGGGCAGCGTGATGAACCAGGTGCGCAACATGATGCTGGTCACCGTCTGGGTGTTCATCGGCATCGAGGGCGCG
AGCATCTTCTCCTCCCGCGCGGAAAAACGTTCCGACGTCGGCAAGGCCACCGTGATCGGCTTCATCACCGTCCTGCTCCTGCTGG
TGCTGGTCAACGTGCTGTCCATGGGCGTGATGACCCAGCCGGAACTGGCCAAGCTGCAGAACCCGTCGATGGCGCTGGTACTCG
AGCATGTGGTCGGCCACTGGGGCGCCGTGCTGATCAGCGTCGGCCTGCTGATCTCGCTGCTGGGCGCGCTGCTCTCCTGGGTGC
TGCTGTGCGCCGAGATCATGTTCGCCGCCGCCAAGGACCACACCATGCCGGAGTTCCTGCGCCGCGAGAACGCCAACCAGGTGC
CGGCCAACGCCCTGTGGCTGACCAACATCTGCGTACAGGTGTTCCTGGTGGTGGTGTTCTTCACCTCGGGCGACCCGGACGGCAT
GGACCCGTACACCAAGATGCTGCTCCTGGCCACCTCGATGATCCTGATCCCGTACTTCTGGTCCGCCGCCTATGGCCTGCTGCTG
ACCCTGAAGGGCGAGACCTACGAGAACGATGCCCGCGAACGCAGCAAGGACCTGGTCATCGCCGGCATCGCCGTGGCCTACGCG
GTCTGGCTGCTCTACGCGGGGGGCCTGAAGTACCTGCTGCTGTCCGCCCTGCTGTATGCGCCCGGCGCGATCCTTTTCGCCAAG
GCCAAGCACGAGGTCGGCCAGCCGATCTTCACCGGAATCGAAAAACTGATCTTCGCCGCAGTCGTCATTGGCGCCCTGGTGGCTG
CCTACGGCCTCTACGACGGTTTCCTCACCCTCGCATTAGAAGTTTTGTTTCAAGGTCCACACCATCATCATCACCATCATCATCATCA
TTAA3’

pNZ*arcD-RF* (*arcD Rhizobium fredii* HH103)

5’CTAGTCTTATAACTATACTGACAATAGAAACATTAACAAATCTAAAACAGTCTTAATTCTATCTTGAGAAAGTATTGGTAATAATATTA
TTGTCGATAACGCGAGCATAATAAACGGCTCTGATTAAATTCTGAAGTTTGTTAGATACAATGATTTCGTTCGAAGGAACTACAAAAT
AAATTATAAGGAGGCACTCACCATGAGTACATCAACTGCTCAAAAACTTAGTCTTGCTTCTTTATCTGCTTTAGTAGTAGGTTCAATG
GTTGGTGCTGGAATTTTTAGTCTTCCACGTACATTTGGAAATGCTACTGGTCCTTTTGGAGCAATTGTTGCTTGGTGTATTGCTGGTG
CAGGAATGTTTACACTTGCACATGTTTTTCGTGTTCTTGCTGAAAGAAAACCAGATTTAGATGCAGGAGTTTATGCTTATGCAAAAGC
TGGTTTTGGAGATTATGCAGGATTTTTATCAGCTCTTGGATATTGGTTAGTTGGTTGTATTGCTGATGTTTCTTATTGGGTTCTTATTA
AAGCAACACTTGGTGCTTTCTTTCCTATTTTTGGTGATGGAAATACTGTTGCTGCAGTTTTAGTTTCATCAGTTGCACTTTGGGGATTT

CATTTTATGATTCTTCGTGGTATTAAAGAAGCTGCAGCTATTAATACAGTTGTTACTGTTGCTAAAATTGTTCCAATTGTTATTTTTATT
GTTATTCTTTTAGGAGCATTTGAAACAGATTTATTTCGTGCTAATTTTTGGGGTGGAGCAGGAATGCCTGAAGCTTCATTATTTGAAC
AAGTTAGAGCAACAATGCTTGTTACTGTTTTTGTTTTTATTGGTGTTGAAGGAGCAAGTGTTTATTCAAGATATGCTCGTAAAAGATCT
GATGTTGGAGTTGCTACAACTCTTGGTTTTGTTGGAGTTCTTGGTTTAATGGTTCTTGTTACTCTTCTTCCATATGCAGCTTTAGAAC
GTCCTGAAATTGCTGGTATGAGACAACCATCTTTAGCTAGTGTTTTAGAAAGTGTTGTTGGACCTTGGGGTTCAGTTTTTGTTTCTGT
TGGATTAATTGTTTCTGTTCTTGGTGCATATTTAGCTTGGAGTTTAATTTGTGTTGAAGTTTTATTTTGTGCAGCTAAAAATGGAGATA
TGCCAAGTGTTCTTGCACGTGAAAATAATAATAATGTTCCTGCAGCTGCATTATGGTTATCAAATGGTGTTATTCAATTTTTCTTAGTT
TCAACATTATTTTCAGAAAATGCATTTAGACTTATGGTTAATCTTACTTCTGCTATGGTTCTTGTTCCATATTTATTAGTTGCTGCATAT
GGATTTTTAATTGCACGTAGAGGTGAAACATATCATATTCGTCCTGAAGAAAGATTTTCTGATCTTATTTTTGCTGGAGCTGCAACAG
TTTATACTGCATTTATGATTTATGCAGGTGGTTTAGAACTTCTTCTTCTTAGTACTATTTTATATGCTTTTGGTACAGTTTTATTTTTATA
TACTCGTAGAGAACAAAAGAAACCATTATTTAATCCTAGAGAATGGCTTGTTTTTATGGCTGCTGCTGTAGGTTGTGTTTTTGGTATTT
ATTCATTAGTTACTGGTTATATTACTTTAGCATTAGAAGTTTTGTTTCAAGGTCCACACCATCATCATCACCATCATCATCATCATTAA3
’

pNZ*arcD-RD* (*arcD Roseobacter denitrificans* ATCC 33942)

5’CTAGTCTTATAACTATACTGACAATAGAAACATTAACAAATCTAAAACAGTCTTAATTCTATCTTGAGAAAGTATTGGTAATAATATTA
TTGTCGATAACGCGAGCATAATAAACGGCTCTGATTAAATTCTGAAGTTTGTTAGATACAATGATTTCGTTCGAAGGAACTACAAAAT
AAATTATAAGGAGGCACTCACCATGAGTGATGATTGCCGGAACCGGAATGTACATGCTGGCGCGTGTGTTTCAGTTTCTGGCGCAG
CGACGTCCCAATCTGGATTCGGGCGTTTTTGCTACGCCAAGGCGGGTTTTGGCAGCTATGTGGGCTTTTTATCTGCATCTGGCTAT
GGGGCCAGCGCGATGCTCGGCAACGTGTTTTACTGGGTGTTGATCAGTGCGACGTTAAGCCTTTTCTTCCCCGCCTTTGGTGACG
GCGGTACAATTCTCGGGATTGTCGTTTCGCTCATCGGCATATGGACGTTCCACTTTATCATTCTGCGGGGCATCCAAGAAGCGGCT
TTCATCAACACTGTTGTTTCAATCGCCAAACTTGTCCCGCTTTTCGTGGCGATAACCGCGATGGTTTTTGTGTTCAACTGGGACACAT
TCAGCGCAAATTTCTGGGGCGGGGCCGACATGCCCGAGAAGTCACTGCTCCTTCAGGTGCGGGATACGATGCTGATCACGGTCTT
CGTTTTCATCGGCATTGAAGGCGCAAGTGTCTATTCACGGTATGCCAAAACACGTCGGGACGTCGGCAGCGCGACCATCTTGGGTT
TTGTCGGCGTGTTGGGCATCATGGTCGCCATCACGCTTTTGCCCTATGGAATCATGCCCCAGTCCGCGGTGGCTGATCTGCGCAA
GCCCTCCCTTGCAGGCGCTTTAGAGGCCACTGTGGGCGCGTTGGAAACCCTGTTCATCTCAATCGGAGTTCTGATTTCGGTTCTGG
GCGCTTATCTTACCTGGTCGCTGCTGGTTGCCGAAGTGCCTTACGCCGCCGCGAAATCAAAGGATATGCCAAGCGTGTTCGGACG
CGAGAACGCGCAAAATGTGCCGGCGAATTCACTGTGGCTCTCGAACAGTATCATATCAGTTTTTCTGATCTCGACCTATTGGTCCGC
TGATGCGTTCAACTTCATGCTGGACATGGCGACCGTCGCATCTCTACTCCCATACTCGCTGGTCGCTGGCTACGGCATCCTTCTCA
CCCGTTCCAACGAGATTTATGGCGATGAACAGGGCAAACAGAAACGCGACAGTATTTTCGCATGGGTCGCCGCGGTCTATGTGGT
GTTCATGTTCGTCGCTGCAGGGTTGAAATACATCATCCTGGTGGCGGTCATCTATGCGCCTCTGACCGTTCTCTACTTTTGGGCAC
GTAAGGAACAAAACCTGCCTTACTTCACAAAAGTGGAATTGATCGTTTTCGGCATCGTTCTTCTAGTGGCCGCTATCGGCGTCTATG
GCCTTTTTTCGGGTGCTATCACTCCTGCATTAGAAGTTTTGTTTCAAGGTCCACACCATCATCATCACCATCATCATCATCATTAA3’

pNZ*arcE-LB* (*arcE Lactobacillus brevis* ATCC 367)

5’CTAGTCTTATAACTATACTGACAATAGAAACATTAACAAATCTAAAACAGTCTTAATTCTATCTTGAGAAAGTATTGGTAATAATATTA
TTGTCGATAACGCGAGCATAATAAACGGCTCTGATTAAATTCTGAAGTTTGTTAGATACAATGATTTCGTTCGAAGGAACTACAAAAT
AAATTATAAGGAGGCACTCACCATGAGTGCTAATGCTGAAGTTCAACCACCGAAGAAGAAATTTAAGCTTAAAATGCCGGGGGCTTT
TGTGATTCTGTTCATTTTAACGATTCTTGCTGTTATGGCAACTTGGGTCATTCCGGCTGGGAGTTATGCAAAACTAACGTATAACCAG
GCGACATCGAGTTTACAAATTAAAAGTCCCACTGGGAAAACAACTCGAGCACCAGCCACCCAAAAAGAATTAGATCGCCTAGGCGT
TAAAATTAATATTGATCAATTTACGTCAGGGGGAATTTCTGAAGCCGTTTCAATTCCCAATACGTATCAACGTTTGAAGCAACGACCG
GCTAGTCTATGGGCCGTAACTGGCAGTATGGTGCGCGGAACTATTGAAGCAGTTGATATCATGGTCTTTATCTTTGTTTTAGGTGGC
CTGATCGGGGTCGTTAAAGCTAGTGGAGCTTTTGAATCGGGACTGATGGCGCTGACCAAAAAGACGAAAGGCCATGAGTTCCTCCT
CATTTTTCTGGTAGCTATTCTGATGGTGCTGGGCGGAACGCTGTGTGGGATTGAAGAAGAAGCAGTAGCCTTTTATCCCATTTTAGT
GCCAATCTTCATTGCAATGGGCTACGATTCAATCGTCTGTGTTGGGGCAATCTTTTTAGCAAGTTCAGTCGGGACAGCCTTTTCAAC
GATTAACCCCTTTTCTGCGGTTATCGCGTCCAATGCTGCTGGAATTGATTTTACGCAAGGGATTGTTTGGCGAATTATCGGCTTGAT
CGTCGCGGCGATCTTCCTTATTTTCTACTTGTATTGGTATAGCAAAAAGGTAAAGAATCAACCAACTTTTTCATACACGTATGAGGAT
CGTGAATCGTTTAATCATATGTGGTCAGTAGCTTCTACGAACGATCAAATTAAGACCGGTTTCTCCATGCGAAAGAAATTGATTTTAA
CATTATTCGTTGTGACCTTCCCAATTATGGTTTGGGGAGTTATGTCACAAGGCTGGTGGTTCCCAACCATGGCCTCGTCATTCTTGG
TGTTTGCTATTATTATTATGTTTTTAACAGCAACGGGTCCTAATGGCTTAGGAGAAAAAGGAGTCGTTGATGCCTTTGTTGCGGGTGC
CTCTAGCCTAGTAGGTGTTTCATTAATCATTGGGTTAGCCCGAGGAATCAATTTAGTTATGAACGAGGGCTTAATTTCCGACACAATT
TTACAATATTTCTCGTCGCTGGTTATTCATGTGAGTGGCCCAATCTTTATTCTTGTCTTGATGTTTATATTCTTTATACTCGGTTTCATT
GTGCCATCTTCATCTGGATTGGCTGTGTTAGCAATGCCAATCTTCGCACCATTAGCGGATACCGTCAACATTCCACGGTTTGCTGTC
GTAACGGCTTATCAGTTTGGACAATATGCCATGCTTTTCTTAGCGCCAACTGGTTTGGTGATGGCAACCTTGCAGATGTTGGACATG
AAGTATTCTCACTGGTTTAAATTTGTTTGGCCAGTAGTCGTATTTGTCCTCACCTTTGGTGGGATACTGTTGGTATTAGAAGTATTAAT
TTCAGGTGTTGCATTAGAAGTTTTGTTTCAAGGTCCACACCATCATCATCACCATCATCATCATCATTAA3’

pNZ*arcE-LS* (*arcE Lactobacillus sakei* ATCC15521)

5’CTAGTCTTATAACTATACTGACAATAGAAACATTAACAAATCTAAAACAGTCTTAATTCTATCTTGAGAAAGTATTGGTAATAATATTA
TTGTCGATAACGCGAGCATAATAAACGGCTCTGATTAAATTCTGAAGTTTGTTAGATACAATGATTTCGTTCGAAGGAACTACAAAAT
AAATTATAAGGAGGCACTCACCATGAGTGATGATGCTGAAATAATCAAGACTAAGAAGCGGTTTAAACTTAAAATGCCGGGGGCTTT
TGTGATCCTCTTCATACTGACAGTGGTCGCGGTGATGGCGACGTGGGTTGTGCCGTCAGGATCTTACGCGAAGTTATCGTATGACC
AAAAGAGTACGCAACTGGTCGTGACAAAACCATCAGGTCAAGTTGAAAAGGTGCCAGCAACACAGGCATCATTAGACCGACTCGGC

GTTAAAATCAAAATTAGTCAATTTACTTCTGGTAGTATTAATGCAGCCGTGTCGATTCCCAATACCTACCAAAGATTGAAACAACGGC
CGGCCAGTTTAGCAGCTGTTCCTAACAGTATGGTTCGTGGGACCGTTGAAGCTGTCGATATTATGGTTTTTATTTTAGTATTAGGGG
GCTTGATTGGCACGGTCAAGGCTAGCGGCGCATTTGAGTCGGGGCTGTTGGCCTTAACCAAGAAAACAAAAGGGCATGAATTTCTG
TTAATCTTTTTCGTCGCGATTCTCATGGTTCTAGGCGGGACTTTATGTGGGATTGAAGAAGAGGCGGTGGCCTTTTATCCAATTTTA
GTACCGGTCTTTATTGCGATGGGGTACGATTCGATTGTTTGTGTGGGGGCGATTTTCCTTGCCAGTTCAATTGGCACGACTTTCTCG
ACCATTAATCCCTTTTCAGTTGTGATTGCTTCAAATGCCGCTGGGATTAGTTTCACAGAAGGCTTGTTGTGGCGAGTTGGCGGCTGT
ATCGTAGGGGCCATTTTTGTCATTTATTATCTCTATCGCTATTCTAAAAAGGTCAAGGCTGATCCGACCCAATCGTATTCATATGAAG
ATCACGACGCTTTTGATAAGATGTGGGCAATTACATCTGAGGGGACGCAGTCAAAAGCCGTTTTTACCTGGCGGAAGAAATTAATTC
TGGTCTTATTTGTGGTGACCTTCCCGATTATGGTCTGGGGCGTTATGTCTCAAGGGTGGTGGTTCCCAACGATGGCGGCGTCATTT
TTAACGTTTGCGATTATCATCATGTTTTTAACCGCGACAGGACCAGAAGGCATTGGCGAAAAAGGGGTTATCGACGCTTTTGTGAAC
GGTGCTTCTAGTTTAGTAGGCGTCTCGCTCATTATTGGTCTTGCGCGAGGGATTAACTTGATTATGAATGAAGGGATGATTTCAGAT
ACCTTATTACAATATTCGTCATCATTAGTCGCACATGTTAGTGGTCCGATTTTTATTTTGATCATGTTAGTGATATTCTTCGTGCTTGG
CTTTATCGTTCCATCTTCTTCGGGACTAGCCGTCTTATCGATGCCGATTTTAGCACCGTTAGCAGATACGGTTAATATTCCCCGATTT
GTTGTGGTAACGGCCTATCAATTTGGGCAATACGCAATGTTATTCTTGGCACCAACTGGCTTGGTAATGGCCACCTTACAAATGTTA
GATGTTAAATACGCTCACTGGTTACGGTTCGTTTGGCCAGTAGTCGCTTTTGTGCTGACTTTTGGTGGGGCCATGTTAGTCTTACAA
GTCCTAATTTATTCGGCATTAGAAGTTTTGTTTCAAGGTCCACACCATCATCATCACCATCATCATCATCATTAA3’

pNZ*arcE-SP* (*arcE Streptococcus pneumoniae* D39)

5’CTAGTCTTATAACTATACTGACAATAGAAACATTAACAAATCTAAAACAGTCTTAATTCTATCTTGAGAAAGTATTGGTAATAATATTA
TTGTCGATAACGCGAGCATAATAAACGGCTCTGATTAAATTCTGAAGTTTGTTAGATACAATGATTTCGTTCGAAGGAACTACAAAAT
AAATTATAAGGAGGCACTCACCATGCACCATCATCATCACCATCATCATCATCATTTAGAAGTTTTGTTTCAAGGTCCAAGTAGTGAA
AAAGCTAAAAAAGGGTTTAAGATGCCTTCATCTTACACCGTATTATTGATAATCATTGCTATTATGGCAGTGCTAACTTGGTTTATCCC
TGCGGGGGCCTTTATAGAAGGTATTTACGAGACTCAGCCTCAAAATCCACAAGGGATTTGGGATGTCCTCATGGCACCGATTCGGG
CTATGCTAGGTACTCATCCAGAGGAAGGTTCGCTCATTAAAGAAACGAGCGCAGCGATTGATGTAGCCTTCTTCATCCTTATGGTTG
GGGGTTTCCTTGGCATTGTCAACAAAACTGGTGCTCTTGACGTAGGGATTGCCTCTATCGTGAAGAAGTATAAGGGCCGCGAAAAA
ATGTTAATTTTGGTACTGATGCCTTTGTTTGCCCTCGGTGGTACAACTTATGGTATGGGGGAAGAAACAATGGCCTTCTATCCACTC
CTTGTGCCAGTTATGATGGCCGTTGGTTTTGATAGCCTGACTGGTGTTGCAATTATTTTGCTCGGTTCTCAAATCGGCTGTTTGGCA
TCTACTCTGAATCCATTTGCGACAGGTATTGCTTCAGCGACTGCGGGAGTTGGTACAGGGGACGGTATCGTACTTCGTCTGATCTT
CTGGGTTACCTTGACTGCTCTTAGTACTTGGTTTGTTTACCGTTATGCGGATAAGATTCAAAAAGATCCGACTAAGTCACTGGTTTAT
AGTACTCGCAAAGAAGATTTGAAACACTTTAACGTAGAAGAATCTTCATCTGTAGAATCTACACTTAGCAGCAAACAAAAATCAGTTC
TCTTCTTATTTGTGTTGACATTCATTTTGATGGTATTGAGCTTCATTCCATGGACAGACCTTGGCGTTACCATTTTTGATGACTTTAAT
GCTTGGTTGACTGGTCTTCCAGTTATTGGTAATATTGTCGGTTCATCTACTTCTGCACTAGGTACTTGGTACTTCCCAGAAGGCGCA
ATGCTCTTTGCCTTTATGGGTATCCTGATTGGTGTTATTTATGGTCTTAAAGAAGATAAGATTATCTCTTCCTTCATGAATGGTGCTGC
TGACTTGCTCAGTGTTGCCTTGATCGTAGCGATTGCTCGTGGTATTCAAGTTATCATGAACGACGGTATGATTACCGATACAATCCT
CAACTGGGGTAAAGAAGGCTTGAGCGGTCTATCTTCACAAGTCTTTATCGTTGTAACTTATATCTTCTATCTACCTATGTCATTCTTG
ATCCCATCTTCATCTGGTCTTGCCAGCGCAACTATGGGTATCATGGCTCCACTTGGAGAATTTGTAAATGTCCGTCCTAGCTTGATT
ATCACTGCTTACCAATCTGCTTCAGGTGTCTTGAACTTGATTGCACCAACATCTGGTATTGTGATGGGAGCTCTTGCACTTGGACGT
ATCAACATTGGTACTTGGTGGAAATTCATGGGCAAACTCGTAGTCGCTATTATTGTAGTGACCATCGCCCTTCTTCTCCTTGGAACCT
TCCTTCCATTCCTATAA3’

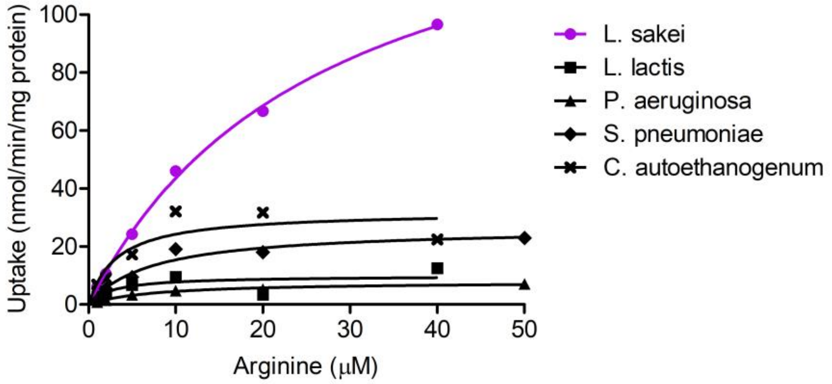

Figure S4. *In vivo* L-arginine uptake by ArcD and ArcE transport proteins. Single colonies of *L. lactis* JP9000 Δ*arcD1*Δ*arcD2* carrying pNZ*arcD/E* were resuspended in 10 mL of M17 medium supplemented with 1% glucose plus 5 µg/mL of chloramphenicol (GM17Cm). This suspension was used to prepare eight precultures by 1:3 *v/v* or 1:12 *v/v* serial dilution into 3 mL fresh GM17Cm that were incubated overnight at 30 ºC without shaking. The following day, 50 mL cultures were prepared by diluting 1:3 *v/v* the overnight pre-cultures, which were in the late exponential phase of growth (0.7 < OD_{600} < 1.5); cultures in the stationary phase (OD_{600} > 2) were diluted 1:100 *v/v*. These cultures were incubated at 30 ºC, 100 rpm, and over-expression was induced at an OD_{600} of 0.4-0.5 with 0.5 ng/mL nisin A. After 1 hour induction, cells were harvested by centrifugation (10 min, 4.000 g, 4 ºC), washed with 50 mM K-HEPES pH 7.0 and resuspended to a final OD_{600} of 50 in 50 mM K-HEPES pH 7.0 supplemented with 1 mM L-ornithine. The resuspended cells were incubated in the presence of 1 mM L-ornithine for 1 hour at RT, or overnight at 4 ºC. Next, a 1:20 *v/v* dilution was prepared by pipetting 5 µL cells into 90 µL of 50 mM K-HEPES pH 7.0, followed by a 30 sec incubation at 20 ºC with stirring. L-arginine uptake was initiated by adding 5 µL of radiolabeled L-arginine from appropriate 20x stocks (final concentration 0-50 µM). Cells were incubated at 20 ºC with shaking, and L-arginine uptake was quenched at desired time intervals (10-20 seconds) by addition of 2 mL ice-cold 100 mM LiCl. An initial timepoint was prepared by adding the LiCl solution before L-arginine. The cells were poured onto 0.45 µm nitrocellulose filters (Cytiva), and washed with additional 2 mL of ice-cold 100 mM LiCl. Filters were collected in 2 mL tubes (Eppendorf) to which 2 mL Ultima Gold MW scintillation fluid (PerkinElmer) was added. Total counts were obtained by adding 5 µL L-arginine solution directly onto a filter. Filters were dissolved by vortexing and radioactivity was measured in a Tri-Carb 2800 high-performance liquid scintillation analyzer (Perkin Elmer).

Table S4. Kinetic parameters of ArcD and ArcE proteins based on *in vivo* L-arginine uptake. *In vivo*, ArcD from *L. sakei* transports L-arginine approximately 10-fold faster than ArcD2 from *L. lactis* at 20 ºC in K-HEPES pH 7.0 (see above). Due to the uncertainty in protein expression level in whole cells, the maximal rates of transport (n=1) were intended as initial screening data and were corroborated further by *in vitro* characterization of the proteins.

Organism	Protein	V_{max} (nmol L-arginine/min/mg protein)	K_M (µM)
C. authoetanogenum	ArcD	32.1	3.2
L. lactis	ArcD2	15.8	12.2
L. sakei	ArcD	160	26.8
P. aeruginosa	ArcD	7.9	7.5
S.pneumoniae	ArcE	26.6	7.2

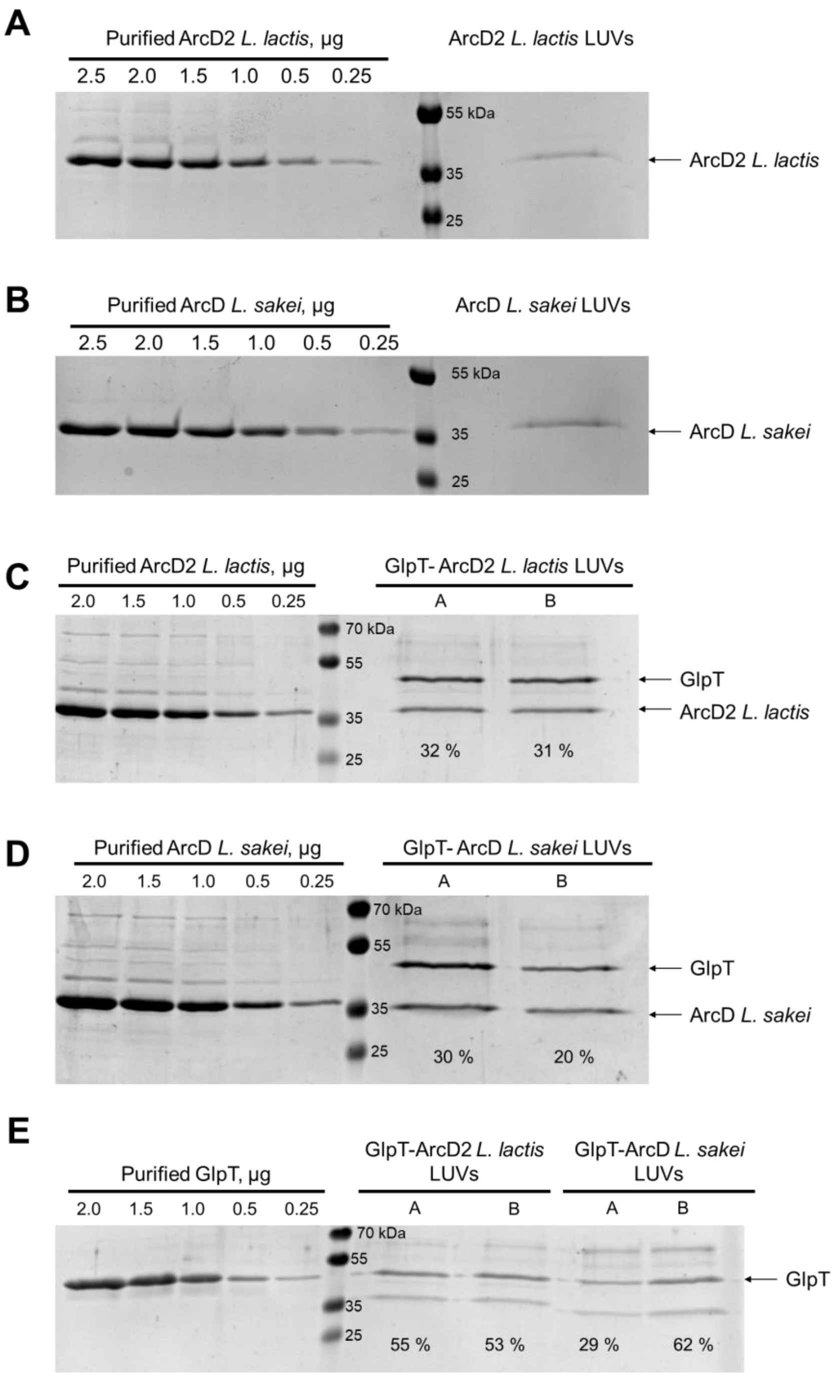

Figure S5. SDS-PAA gel images of purified membrane proteins used in this study. The bands of purified membrane proteins were used to quantify the efficiency of (co-)insertion into vesicles. ArcD2 from *L. lactis* (57 kDa)[1]; ArcD from *L. sakei* (54 kDa); GlpT from *E. coli* (54 kDa)[10]. All proteoliposomes have lipid-to-protein ratios of 400:1 (*w/w*) for each membrane protein. A) Purified ArcD2 from *L. lactis* and proteoliposomes. B) Purified ArcD from *L. sakei* and proteoliposomes. C) Co-reconstitution of ArcD2 from *L. lactis* and GlpT in proteoliposomes. Efficiency of reconstitution of ArcD2 from *L. lactis* when co-reconstituted with GlpT. D) Co-reconstitution of ArcD from *L. sakei* and GlpT in proteoliposomes. Efficiency of reconstitution of ArcD from *L. sakei* when co-reconstituted with GlpT of. E) Purified GlpT and co-reconstitution of ArcD2 from *L. lactis* or ArcD from *L. sakei* and GlpT in proteoliposomes. Bands at ~35 and ~70 kDa represent monomers and dimers of the ArcD proteins, respectively. Efficiency of co-reconstitution of GlpT and either ArcD protein.

Table S5. Kinetic parameters of *L. sakei* ArcD and *E.* coli GlpT. K_M, V_{max} and k_{cat} values of transporters, taken from the literature or determined in this study. Parameters for *L. sakei* ArcD were determined at 30 ºC in DOPE:DOPG:DOPC vesicles (25:25:50 mol%). The kinetic parameters for *E. coli* GlpT were determined at 37 ºC in *E. coli* total lipid extract-egg yolk PC vesicles (3:1 w/w)[12].

Protein	Internal substrate	External substrate	K_M (μM)	V_{max} (μmol x min^{-1} x mg protein^{-1})	k_{cat} (s^{-1})	Ref.
ArcD (*L. sakei*)	L-ornithine (10 mM)	L-arginine	12.2 ± 1.5	6.7 ± 1.4	5.8 ± 1.2	This study
GlpT (*E. coli*)	Inorganic phosphate (Pi, 100 mM)	Glycerol 3-P	164	9.7	8.6	[12]

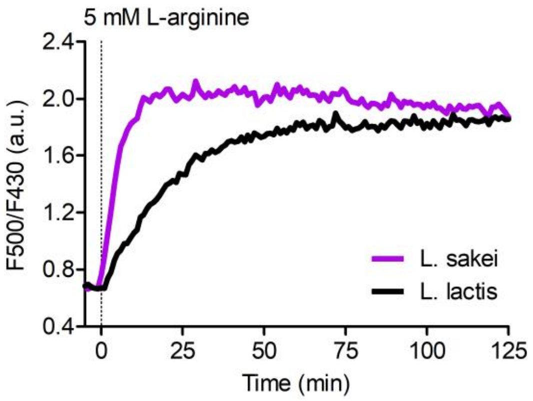

Figure S6. L-arginine-mediated ATP formation in *L. sakei* ArcD or *L. lactis* ArcD2 proteo-LUVs. The ATP/ADP ratio was determined online by acquiring PercevalHR excitation spectra over time from the same proteoliposomes used for ATP quantification with the luciferase assay; traces are representative of at least 3 independent co-reconstitutions. The fluorescence data is in agreement with the chemiluminescence results (see Main text, Figure 3b) and indicates that ATP formation is ~2.5x faster with ArcD from *L. sakei* than with ArcD2 from *L. lactis*.

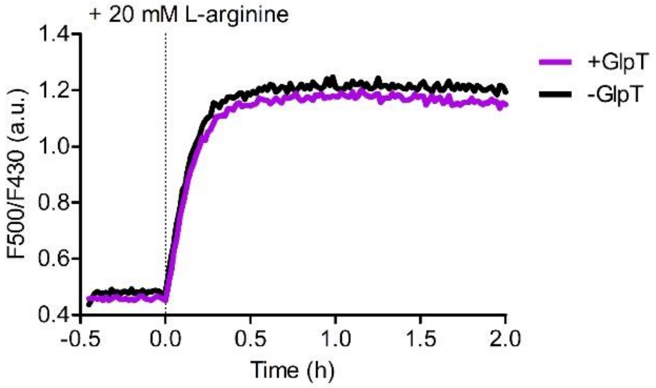

Figure S7. Activity of the feeder vesicles measured by online ATP/ADP readout with PercevalHR. The experiment was carried out as reported in the Main text (see Methods section). Both types of vesicles (with ArcD from *L. sakei* plus GlpT from *E. coli* and the negative control with ArcD from *L. sakei* only) produced ATP upon addition of 20 mM L-arginine, as revealed by the increase in the F500/F430 ratio; traces are representative of at least 3 independent co-reconstitutions.

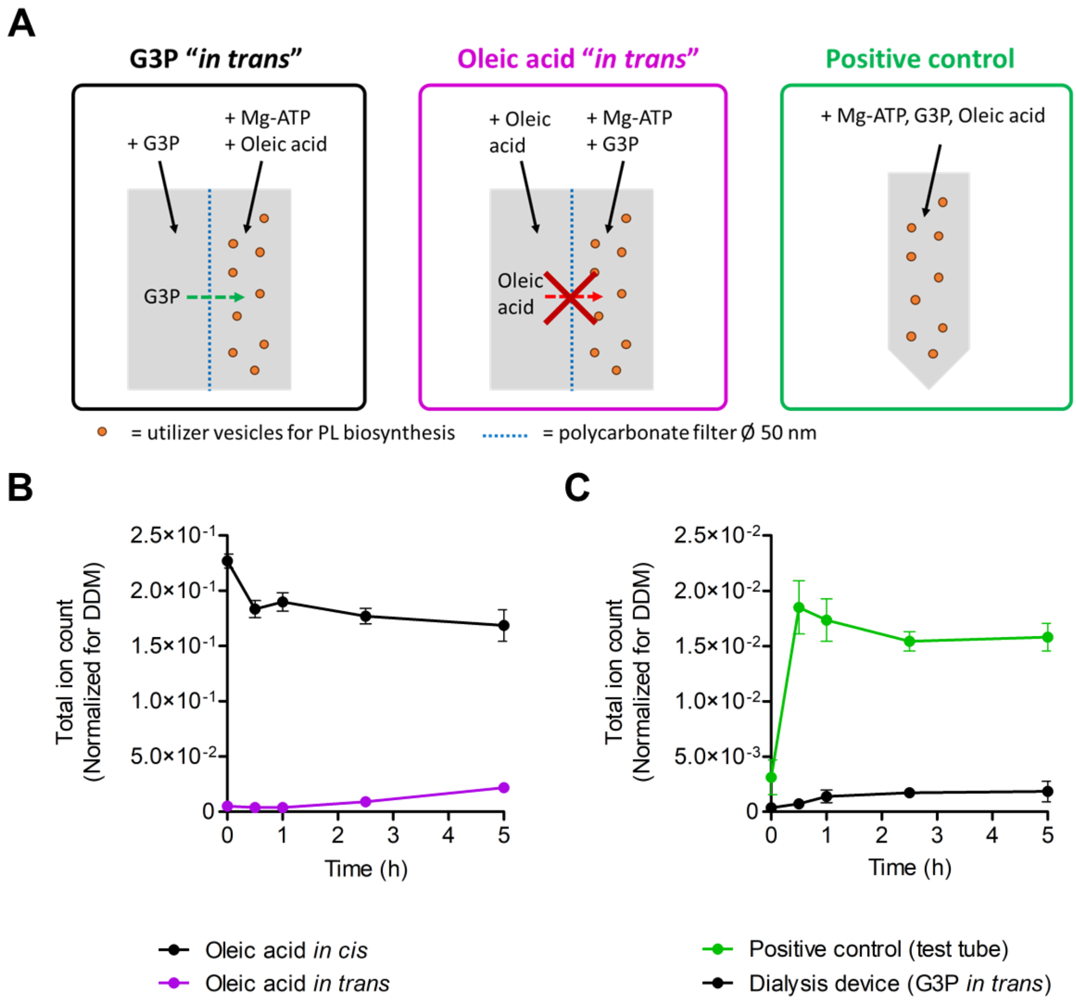

Figure S8. Diffusion of phospholipid precursors through polycarbonate filters. A) Schematic of the experimental setup. Utilizer vesicles (0.8 mL, 2.7 mg/mL total lipids, see Main text, Methods section for preparation) were added to a dialysis chamber equipped with a polycarbonate filter (50 nm pore Ø) and pre-equilibrated with 50 mM KPi, pH 7. To determine whether long-chain fatty acids equilibrate through the dialysis filter, oleic acid (0.8 µM) was added directly to the utilizer vesicles (left panel, black), or to the *trans* compartment (central panel, purple). To determine whether phospholipid biosynthesis is kinetically limited by the slow equilibration of the precursor produced by the feeder vesicles, glycerol 3-phosphate (0.8 µM) was added either directly to the utilizer vesicles (central panel, purple) or *trans* compartment (left panel, black). A positive control was prepared by adding the utilizer vesicles and the precursors of phospholipid biosynthesis to a test tube (right panel, green). A t=0 a 80 µL sample was taken (control), and the reaction was started by addition of Mg-ATP (2 mM) to the utilizer vesicles. Subsequent time points were taken at t=0.5,1,2.5 and 5 hours and analyzed as detailed in the Main text (see Methods section). B) Normalized oleic acid total ion counts (n=3 independent reconstitutions, error bars are s.e.m.). Oleic acid does not equilibrate much across the polycarbonate dialysis device over the 5 hours. The dialysis chamber can thus be used to avoid possible leakage of the feeder vesicles induced by long-chain fatty acids. C) Normalized LPA total ion counts (n=3 independent reconstitutions, error bars are s.e.m.). Diffusion of glycerol 3-phosphate through the polycarbonate dialysis filter is rate-limiting for phospholipid synthesis as can be seen when the green and black lines are compared.

Table S6. Mass-to-charge ratio of lipid species used in this study.

Lipid species	m/z [M-H]$^-$
di-oleoyl phosphatidylglycerol (DOPG)	773.53
di-oleoyl phosphatidylethanolamine (DOPE)	742.54
di-oleoyl phosphatidylcholine (DOPC)	830.59*
di-oleoyl phosphatidic acid (DOPA)	699.49

* Formate adduct [M + CHO2-]$^-$

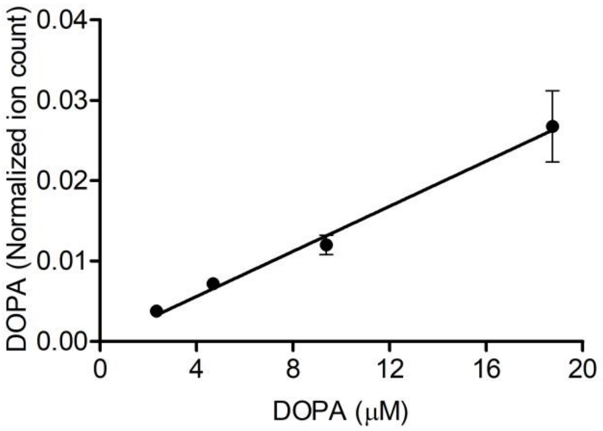

Figure S9. Calibration curve for DOPA quantification. A calibration curve was prepared by mixing 2.3-18.8 mM DOPA with DOPG, DOPE and DOPC (total 3.5 mM, 25:25:50 mol%) from chloroform stocks. The chloroform was evaporated under gaseous nitrogen flow, the lipids resuspended in 50 µL methanol and analyzed by LC-MS as detailed in the Main text (see Methods section). The DOPA total ion counts were normalized for the internal standard DOPG. The calibration curve (n=3-4, error bars represent s.e.m.) was used for quantification of DOPA (normalized for DOPG) from the feeder-utilizer LUVs experiment.

REFERENCES SUPPORTING INFORMATION

1. Pols, T., Singh, S., Deelman-Driessen, C., Gaastra, B. F. & Poolman, B. Enzymology of the pathway for ATP production by arginine breakdown. *FEBS J* **288**, 293–309 (2021).

2. Sievers, F. *et al.* Fast, scalable generation of high-quality protein multiple sequence alignments using Clustal Omega. *Mol Syst Biol* **7**, 539 (2011).

3. Hallgren, J. *et al.* DeepTMHMM predicts alpha and beta transmembrane proteins using deep neural networks. *bioRxiv* (2022) doi:10.1101/2022.04.08.487609.

4. Geertsma, E. R. & Dutzler, R. A Versatile and Efficient High-Throughput Cloning Tool for Structural Biology. *Biochemistry* **50**, 3272–3278 (2011).

5. Geertsma E.R. & Poolman B. High-throughput cloning and expression in recalcitrant bacteria. *Nat Methods* **4**, 705–707 (2007).

6. Majsnerowska, M., Noens, E. E. E., Lolkema, J. S., Henkin, T. M. & Noens, E. E. Arginine and Citrulline Catabolic Pathways Encoded by the arc Gene Cluster of Lactobacillus brevis ATCC 367. *J Bacteriol* **200**, e00182 (2018).

7. Noens, E. E. E. & Lolkema, J. S. Convergent evolution of the arginine deiminase pathway: the ArcD and ArcE arginine/ornithine exchangers. *Microbiologyopen* **6**, e00412 (2017).

8. Pols, T. *et al.* A synthetic metabolic network for physicochemical homeostasis. *Nat Commun* **10**, (2019).

9. Bailoni, E. & Poolman, B. ATP Recycling Fuels Sustainable Glycerol 3-Phosphate Formation in Synthetic Cells Fed by Dynamic Dialysis. *ACS Synth Biol* **11**, 2348–2360 (2022).

10. Auer, M. *et al.* High-Yield Expression and Functional Analysis of Escherichia coli Glycerol-3-phosphate Transporter. *Biochemistry* **40**, 6628–6635 (2001).

11. Exterkate, M., Caforio, A., Stuart, M. C. A. & Driessen, A. J. M. Growing Membranes in Vitro by Continuous Phospholipid Biosynthesis from Free Fatty Acids. *ACS Synth Biol* **7**, 153–165 (2018).

12. Law, C. J., Yang, Q., Soudant, C., Maloney, P. C. & Wang, D.-N. Kinetic Evidence Is Consistent with the Rocker-Switch Mechanism of Membrane Transport by GlpT. *Biochemistry* **46**, 12190–12197 (2007).
